# Supplementary material for: RNA-controlled nucleocytoplasmic shuttling of mRNA decay factors regulates mRNA synthesis and a novel mRNA decay pathway
Source: Nat Commun. 2022 Nov 23;13:7184. doi: 10.1038/s41467-022-34417-z (PMC9684461; doi:10.1038/s41467-022-34417-z)
Supplement: Supplementary file 1 — Supplementary Information [file 41467_2022_34417_MOESM1_ESM.pdf]

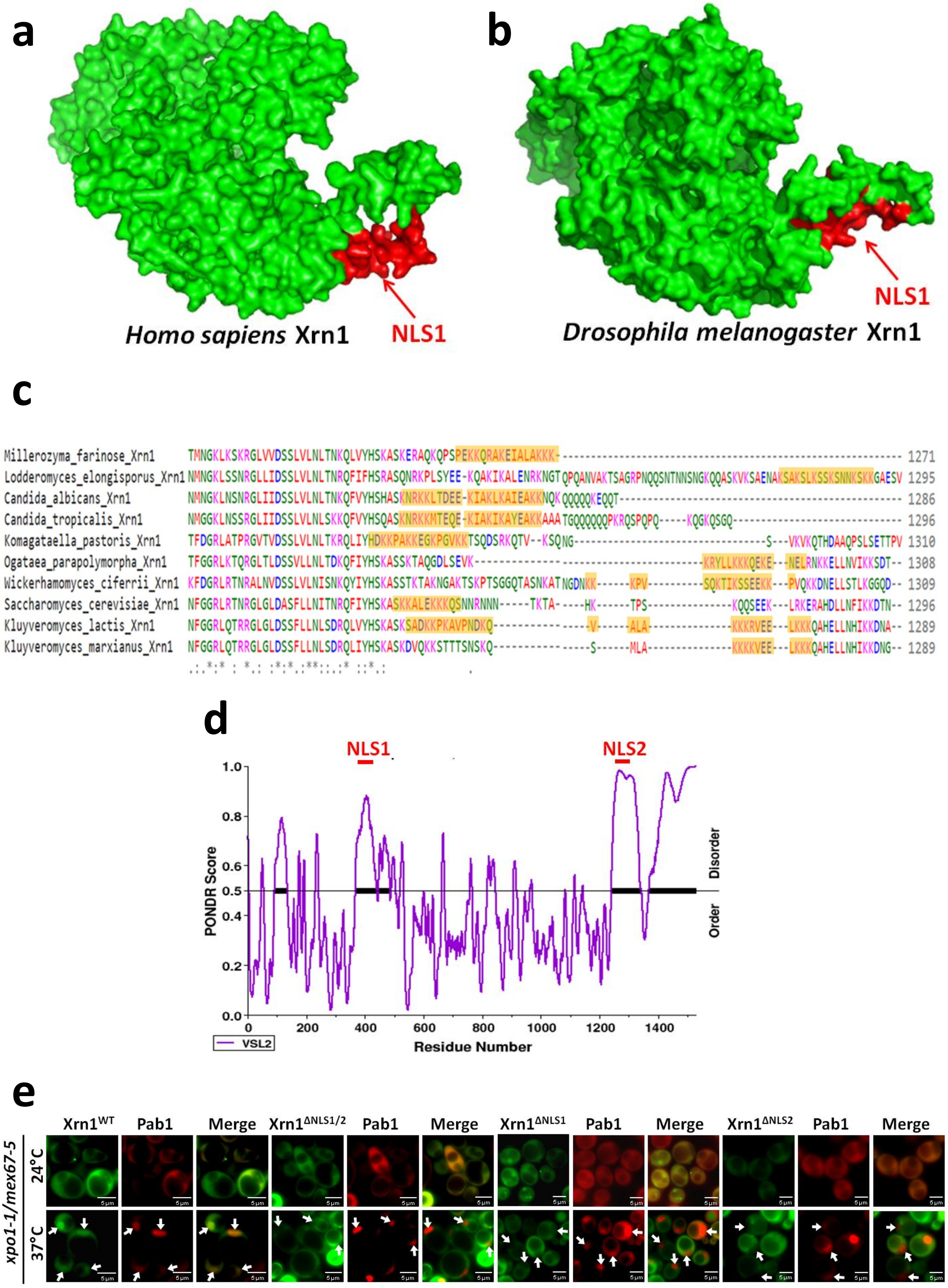

Supplementary Figure 1– p1 of 2

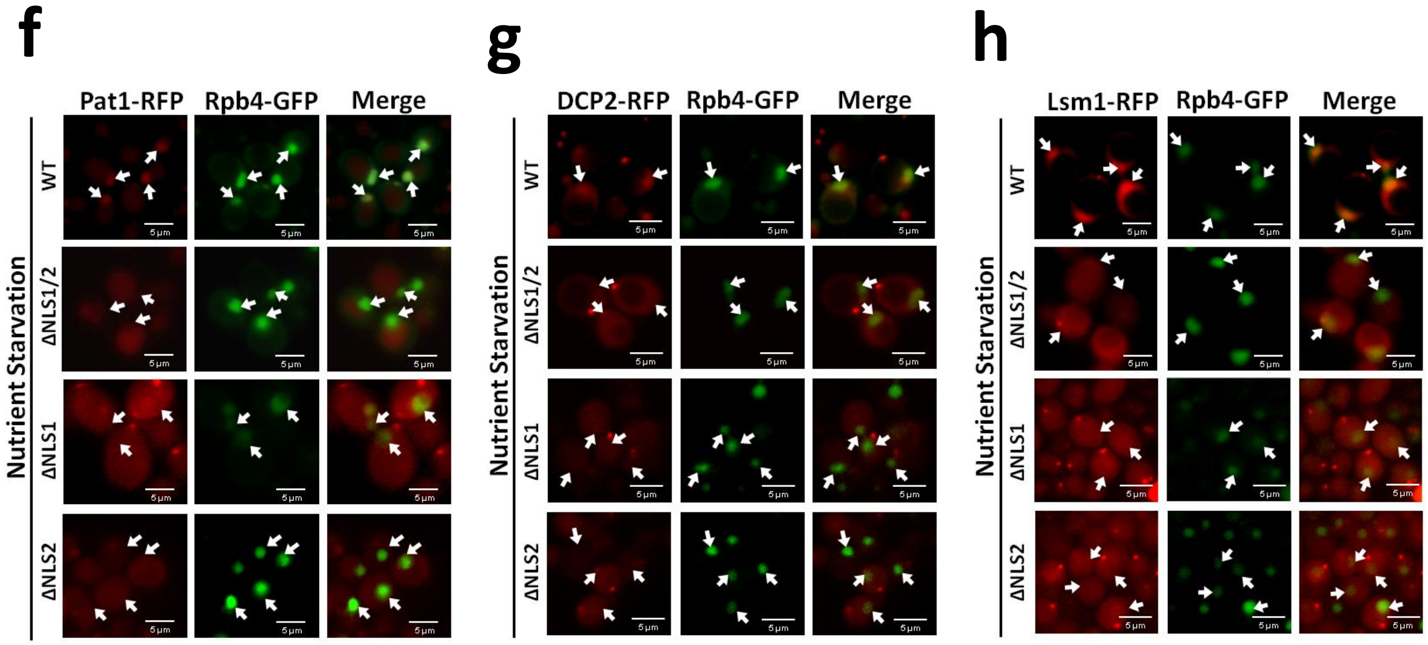

Supplementary Figure 1. Conserved nature and positions of Xrn1 NLSs. Related to Fig. 1. **(a-c)** Higher eukaryotes contain NLS1 in a structurally similar position. **(a) Human Xrn1 NLS1.** Homology model generated by SWISS-model based on the hXrn1 sequence and KlXrn1 structure (PDB ID: 3PIE). **(b)** ***Drosophila melanogaster*** **Xrn1 NLS1.** NLS1, found by cNLSmapper, was highlighted in the model of DmXrn1 based on crystal structure (PDB ID: 2Y35). **(c) Alignment of the region in Xrn1 that contains NLS2 with Xrn1 sequences from several indicated other yeast species.** Alignment was done in Clustal Omega. NLSs were predicted using both cNLSmapper and NLStradamus programs (the two programs made almost the same predictions) and positive results are highlighted in yellow. **(d) Xrn1 NLSs are unstructured.** Disorder plot of Xrn1 was performed using PONDR (http://www.pondr.com/) Program. **(e) NLS1 and NLS2 are required for Xrn1-GFP import.** Import assay of cells co-expressing Pab1p-RFP (served as a positive control and a nuclear marker) and Xrn1-GFP, or its indicated mutant derivatives, was performed as described in Fig. 1c.Arrows point at fluorescently labelled nuclei. Representitive images from 3 biologically independent experiments (n=3). **(f-h) Starvation-induced nuclear localization of the indicated DFs is dependent on Xrn1 NLSs.** WT or the indicated mutant cells expressing the indicated GFP fused DF were shifted from optimal conditions to a starvation medium lacking sugar and amino acids. After 1 h, fluorescence images were taken. Representative images from 3 biologically independent experiments (n=3). Quantifications are shown in Fig. 1e-g.

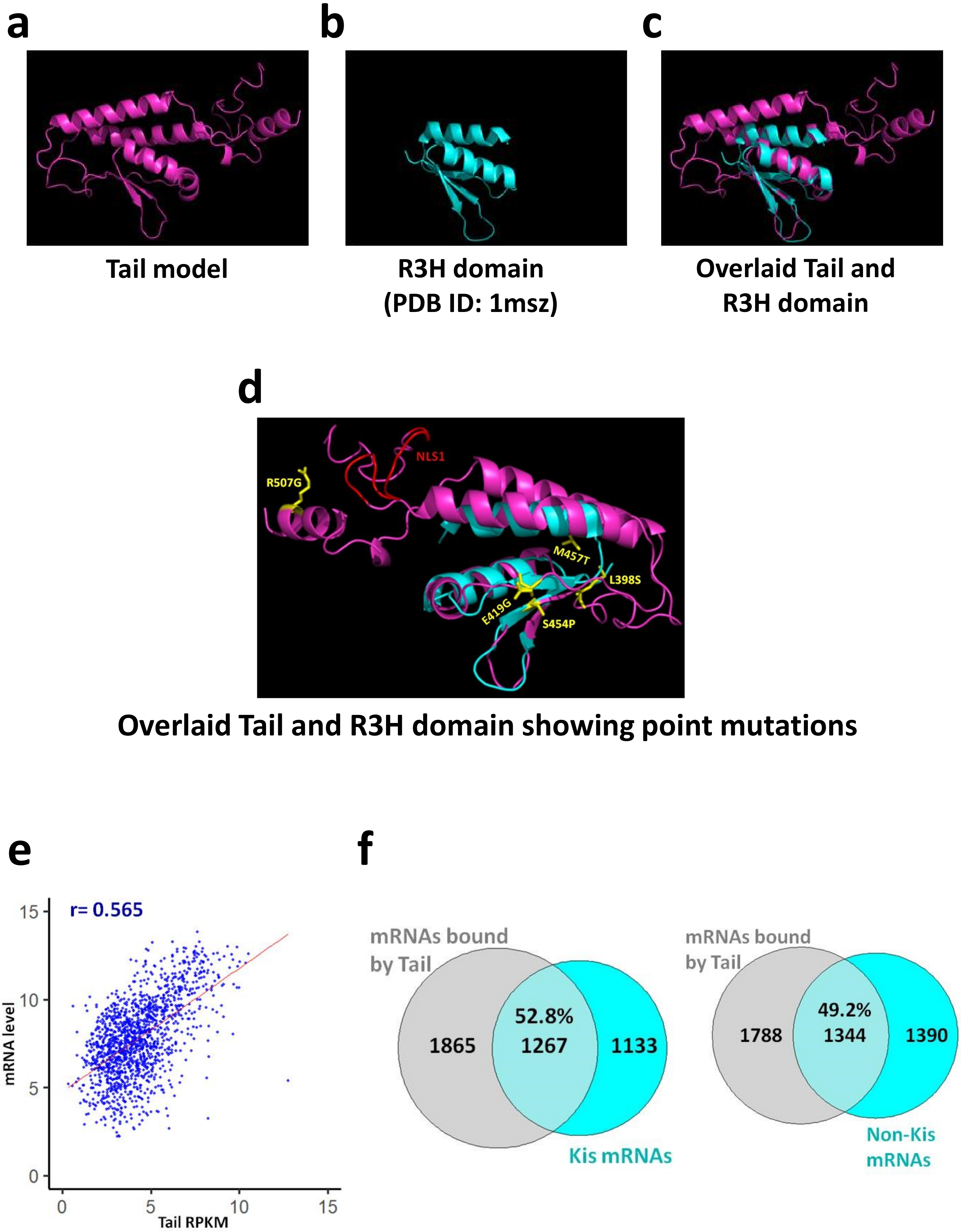

Supplementary Figure 2

Supplementary Figure 2. Mutations that cause constitutive nuclear localization of Tail are mapped within an R3H-like domain. Related to Fig. 2. **(a) Tail homology model was generated by SWISS-MODEL**, using available Xrn1 structure (PDB ID: 6Q8Y). **(b) Smubp-2 R3H RNA binding domain (PDB ID: 1msz). (c) Overlaying of Tail model, based on PyMol, with Smubp-2 R3H nucleic acid binding domain [75].** Homology model of Tail (Purple) is overlaid, using PyMol program, with Smubp-2 R3H RNA binding domain (Cyan). **(d) Different angle of the overlaid model shown in c, indicating, in yellow, the point-mutations that led to Tail-GFP nuclear localization.** We note that the R507G mutation had a modest nuclear localization phenotype. NLS1 is indicated in red. **(e) Binding of Tail to mRNA is correlated with the cellular levels of these mRNAs.** Pearson's Correlation coefficient (r) calculation was performed by "ggpubr" package in R environment. P value of the correlation test is indicated. **(f) Tail, expressed outside the Xrn1 context, binds equally well Kis and non-Kis mRNAs *in vivo*.** Venn diagram between the indicated mRNA groups was perform using the following online resource (http://bioinformatics.psb.ugent.be/webtools/Venn/) and "eulerr" package in R environment. Percent of Tail bound mRNA relative to the total Kis mRNAs (left) or total non-Kis mRNAs (right).

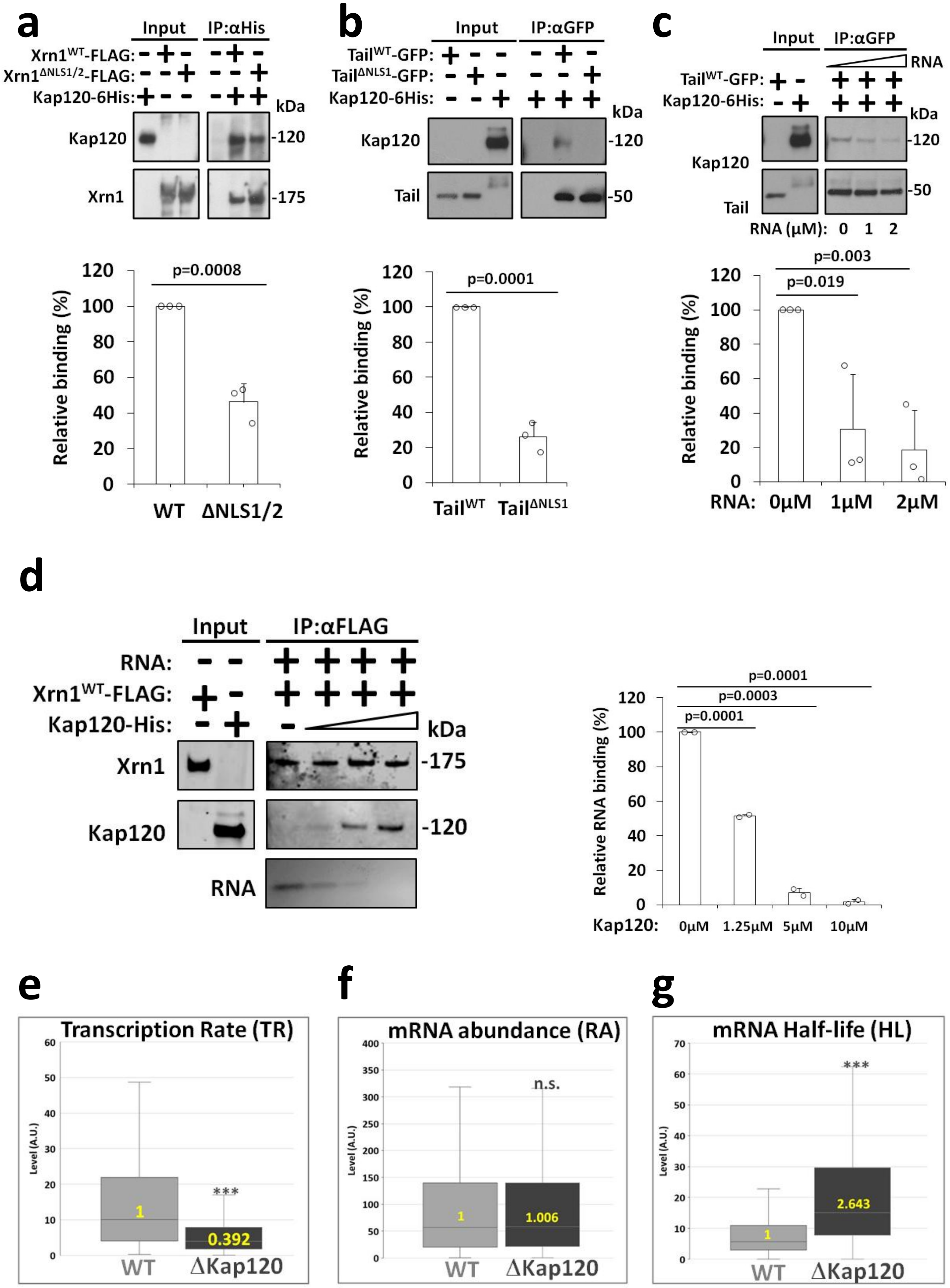

Supplementary Figure 3

Supplementary Figure 3. Tail binds Kap120: the augmenting effect of NLS1 and repressing effect of RNA. Related to Figs. 3 and 4. (a) Interaction ofKap120 with Xrn1 is mediated by its NLSs. Affinity purified Xrn1-FLAG or its mutant derivative, Xrn1ΔNLS1/2-FLAG, and recombinant Kap120-6xHis that had been purified with Ni-NTA column were mixed together, as indicated, for 60 min and co-IPed with anti-FLAG Abs-coupled beads, followed by Western blot analysis using antibodies against the indicated tagged proteins. Bottom panel: Western blot signal of 3 replicates was quantified and plotted. Kap120/Xrn1 ratio of WT is arbitrarily expressed as 100%. n=3 biologically independent samples. Error bars represent standard deviation (S.D.). p-value was calculated using two-tailed Student's unpaired T-test. **(b) Tail binds Kap120 by NLS1-dependent manner.** Tail-GFP or TailΔNLS1-GFP were affinity purified using single chain anti-GFP Abs-coupled beads (GFP-Trap®, Chromotek). Equal amount of Kap120 was then added to the Tail-GFP bound beads and incubated for 60 min. Beads were washed and proteins were eluted by Laemmli sample buffer and boiling, followed by Western blot analysis using Abs against the indicated tagged proteins. Bottom panel: Western blot signal of 3 replicates was quantified. The ratio of Kap120/Tail was arbitrarily defined as 100%. n=3 biologically independent samples. Error bars represent standard deviation (S.D.). p-value was calculated using two-tailed Student's unpaired T-test. **(c) Kap120-Tail interaction is inhibited by RNA. Tail-GFP was purified as in b.** RNA was added to the Tail-GFP-beads and samples were incubated for 20 min at room temperature followed by Kap120 addition as in b. Western blot was performed as in B. Bottom panel: Western blot signal of 3 replicates was quantifies. The ratio of Kap120/Tail in the absence of RNA was arbitrarily defined as 100%.n=3 biologically independent samples.Error bars represent standard deviation (S.D.). p-value was calculated using two-tailed Student's unpaired T-test. The RNA concentrations are indicated at the bottom of the plot. **(d) RNA-Xrn1 interaction is hampered by Kap120.** Xrn1 was purified as in Fig. 3c. Kap120 was added to the Xrn1-FLAG-beads and samples were incubated for 20 min at room temperature followed by addition of Cy3 labeled 40 b RNA. Western blot was performed as in b. Western blot signals of two replicates were quantified and the RNA/Xrn1 signals was determined. The ratio of RNA/Xrn1 in the absence of Kap120 was arbitrarily defined as 100%; n=2 biologically independent samples. Error bars represent standard deviation (S.D.). p-value was calculated using two-tailed Student's unpaired T-test. The Kap120 concentrations are indicated at the bottom of the plot. **(e-g) mRNA abundance (RA), Transcription rate (TR) and Half-life (HL) measurement in Δkap120 cells.** Genomic Run-On (GRO) analysis was performed, in 3 replicates (n=3), as described in Method. Box and whisker plots of the median levels (in arbitrary units) are shown here. Each box represents the 25th to 75th percentile of values, with the median noted by the horizontal bar. Whiskers terminate at maxima/minima or a distance of 1.5 times the IQR away from the upper/lower quartile, whichever is closer; any points correspond to values beyond the latter limit.p-values were obtained by Wilcoxon rank sum test, ***: $p<0.0001$, ns: not significant.

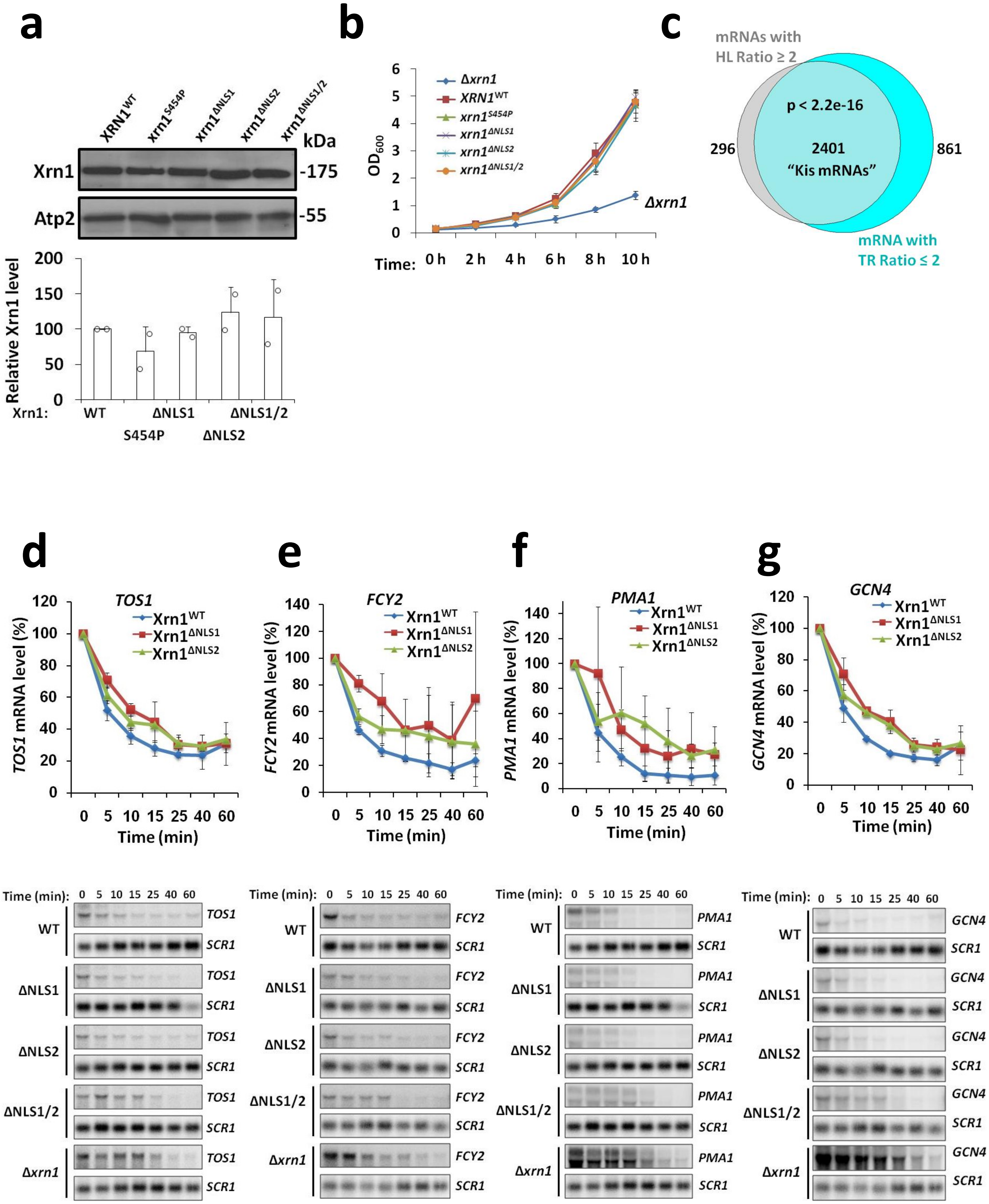

Supplementary Figure 4 – p1 of 2

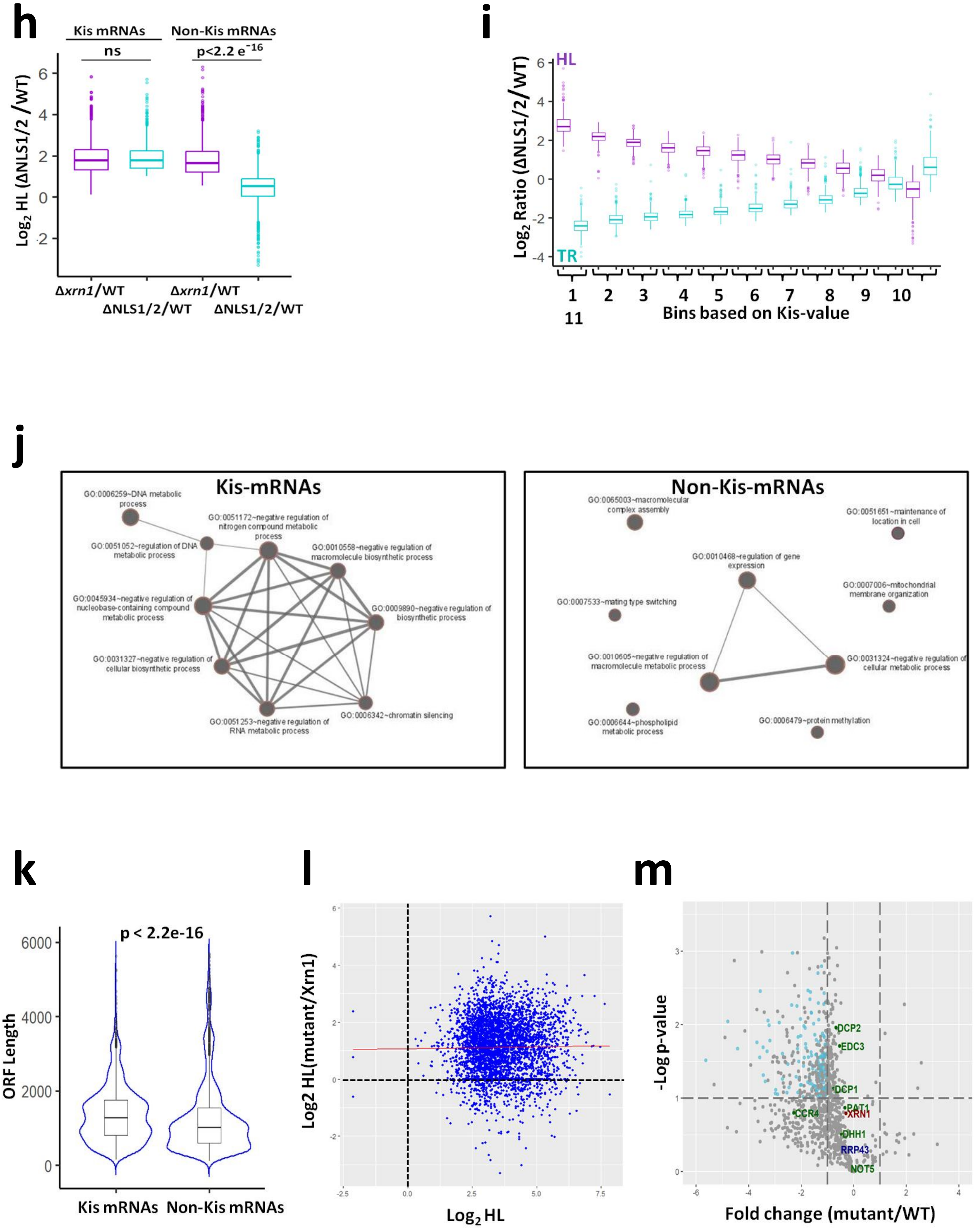

Supplementary Figure 4 – p2 of 2

Supplementary Figure 4. Various features of Xrn1 carrying mutations in its NLSs. (a) Protein levels of WT Xrn1 and its indicated mutant derivatives. Equal amount of whole-cell extracts, taken from optimally proliferating cells expressing FLAG-tagged Xrn1, or its indicated mutant derivatives, were analysed by western blot. Membrane was decorated with anti-FLAG antibody and with anti-ATP2 that was used as a loading control. Lower panel: Quantification of immunoblots. Images were acquired using ImageQuant, quantification of western blot bands were done using TotalLab software. Signal of Xrn1 was normalized to that of Atp2. n=2 biologically independent samples. Error bars represent standard deviation (S.D.). p-value was calculated using Student's unpaired T-test. **(b) Proliferation curve of WT and the indicated xrn1 mutants.** Proliferation curves were determined by monitoring OD_{600} signals. n=2 biologically independent samples.Error bars represent standard deviation (S.D.). p-value was calculated using Student's unpaired T-test. **(c)** Identification of 2401 "Kis-mRNAs".Venn diagram of mRNAs whose TRs decreased ≥2-fold in xrn1$^{\Delta NLS1/2}$ mutant cells relative to WT (right group) and mRNAs whose HLs increased≥2-fold in xrn1$^{\Delta NLS1/2}$ mutant cells relative to WT (left group). Fischer exact test was performed to obtain the indicated p-value. **(d-g) The effect of mutating single NLS on decay rate of specific mRNAs, determined by Northern blot hybridization.** Decay assay of the indicated mRNAs was performed as described in Methods and published previously . Shown are mRNA levels, quantified by PhosphImager and normalized to SCR1 mRNA, as a function of time post-transcription arrest. n=2 biologically independent samples.Error bars represent standard deviation (S.D.) of 2 biologically independent assays. **(h) Effect of xrn1$^{\Delta NLS1/2}$ on Kis mRNAs HLs is comparable to that of XRN1 deletion, whereas it has little effect on HLs of non-Kis mRNAs.** Box and whisker plot of the $\log_2$HLs ratios between mutant and WT, as indicated below the boxes, for Kis and non-Kis mRNAs. Each box is based on 3 replicates. P-values were obtained by Wilcoxon rank sum test comparing WT and xrn1$^{\Delta NLS1/2}$ mutant. Ns – Not significant. Note that the two groups of mRNAs are equally affected by XRN1 deletion. **(i) Kis value represents a gradual spectrum.Genes were arrange according to their Kis value and listed in descending order.** The list was then divided into 11 equal bins. **(j) GO analysis of Kis and non-Kis mRNA products:** Enrichment of the Kis and non-Kis gene groups were done using DAVID. Enriched KEGG pathways were visualized with Cytoscape software. **(k)Violin plot of ORF length of Kis and non-Kis mRNAs.** P-value is indicated. **(l) The effect of Xrn1 import on mRNA HLs is not correlated with the actual HLs in WT cells.** A scatter plot of Log2 fold change of HL in NLS1/2 mutant as a function of HL. Red line represents a trendline of the data. **(m) Protein interactome of Xrn1 compared with that of Xrn1$^{\Delta NLS1/2}$.** Cells expressing FLAG-tagged Xrn1 or Xrn1$^{\Delta NLS1/2}$were subjected to co-immunoprecipitation using Anti-FLAG beads, followed by mass-spectrometry. Raw data were analysed as indicated in Methods. Shown is a volcano plot of WT/mutant ratio. x-axis represents the fold change difference between the WT and the NLS mutant and the y axis represents the -Log(p value). n=2. Several mRNA decay factors were identified and marked.

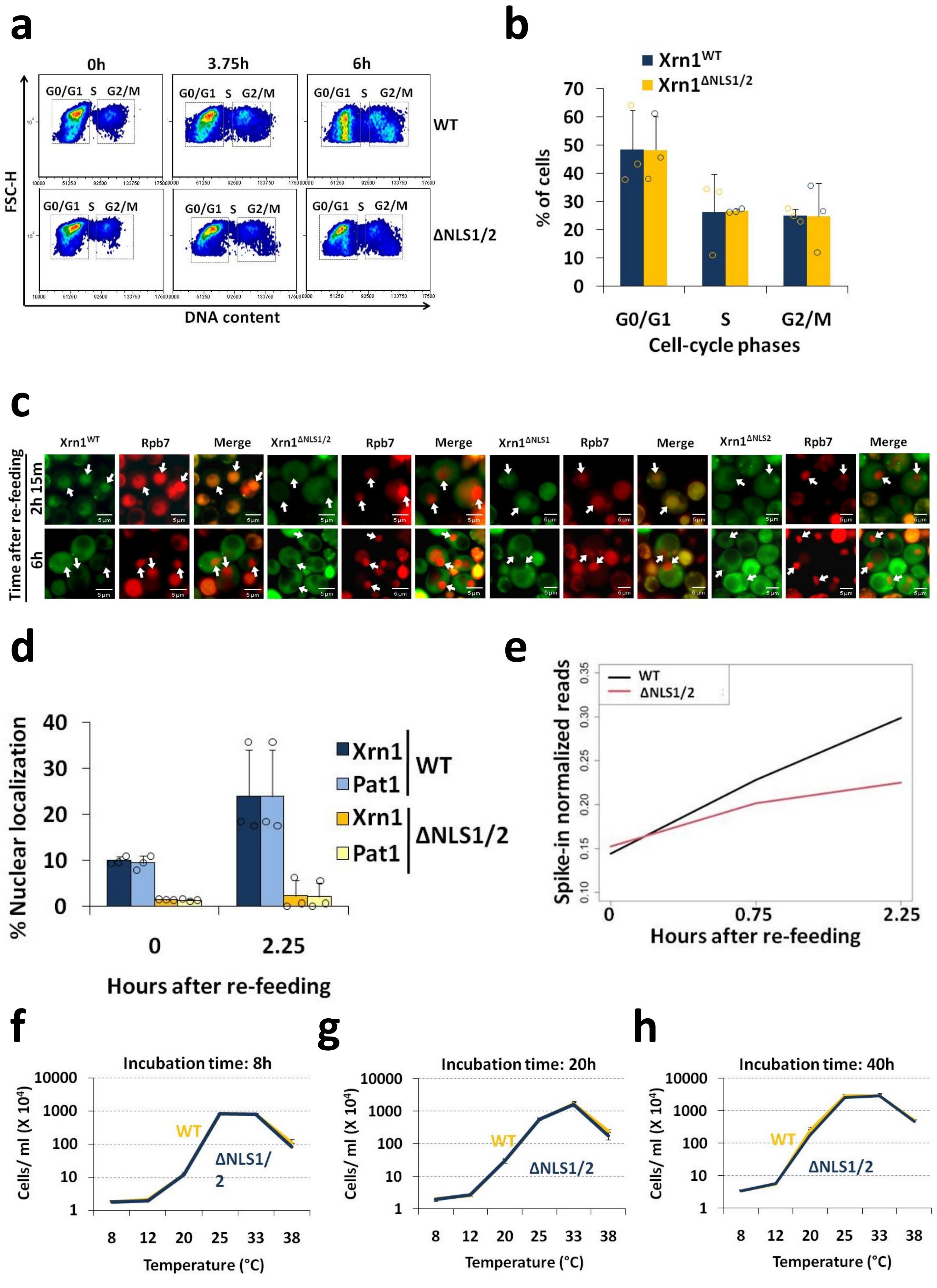

Supplementary Figure 5

Supplementary Figure 5. The involvement of Xrn1 in cell responses to environmental changes. (a) Cell-cycle analysis of WT and $xrn1^{\Delta NLS1/2}$ mutant cells during starvation exit. Optimally proliferating WT or mutant cells, as indicated, were analysed by Flow cytometry (FACS), as in Fig. 6 b-c. Percent of cells with the indicated DNA content (G0/G1, S, G2/M) was determined and plotted at the indicated time points post re-feeding. n=3 biologically independent samples. **(b) DNA content of WT and $xrn1^{\Delta NLS1/2}$ mutant cells under optimal proliferation conditions.** Cells were analysed by Flow cytometry (FACS), as in Fig. 6 B-D. Percent of cells with the indicated DNA content (G0/G1, S, G2/M) was determined and the average of 3 replicates is plotted. Error bars represent S.D. **(c) Cellular localization of the indicated fluorescent protein in starved cells and re-fed cells.** Cells were starved and re-fed, as in Fig. 6d-e, and were inspected under the fluorescent microscope after the indicated time post-re-feeding. The Pol II subunit Rpb7 served as the nuclear marker. Arrows point at some nuclei. Representative images from 3 biologically independent experiments are shown. **(d) During exit from starvation, Pat1 nuclear import depends on Xrn1 NLSs.** Cells expressing GFPfusedXrn1 or $Xrn1^{\Delta NLS1/c2}$ and co-expressing Pat1-RFP were inspected microscopically. Starved cells were re-fed as in Fig. 6e. At the indicated time, the nuclear/whole-cell ratio of the mean fluorescence intensity was determined by ImageJ, as described in Methods. n=3 biologically independent samples.Error bars represent standard deviation (S.D.). **(e) Kinetics of mRNA level increase after re-feeding of starved cells is affected by NLS1/2 disruption.** WT and $xrn1^{\Delta NLS1/2}$ strains that had been starved for 7 days were re-feed by fresh medium. Cells were harvested at the indicated time after re-feeding and mixed with equal number of *S. pombe* cells, as a spike-in. RNA extracted and sequenced. Spike-in normalized reads are shown. **(f-h) Xrn1 import is not required for normal proliferation rate under constant temperatures.** WT or $Xrn1^{\Delta NLS1/2}$cells were allowed to proliferate as in Fig. 6i, except that the temperature remained constant. Gradient PCR machine was used, whereby each tube was incubated at a constant temperature; the different temperatures are indicated in the X-axis. Cell number was measures after 8 h **(f)**, 20 h **(g)** and 40 h **(h)**. n=3 biologically independent samples. Error bars represent standard deviation (S.D.).
