# Supplementary material for: RNA-controlled nucleocytoplasmic shuttling of mRNA decay factors regulates mRNA synthesis and a novel mRNA decay pathway
Source: Nat Commun. 2022 Nov 23;13:7184. doi: 10.1038/s41467-022-34417-z (PMC9684461; doi:10.1038/s41467-022-34417-z)
Supplement: Supplementary file 3 — Description of Additional Supplementary Files [file 41467_2022_34417_MOESM3_ESM.pdf]

Description of Additional Supplementary Files

File Name: Supplementary Data 1
Description: Yeast strains used in the study

File Name: Supplementary Data 2
Description: Plasmids used in the study

File Name: Supplementary Data 3
Description: Genomic run-on (GRO) of delta KAP120 and NLSs mutants experiment

File Name: Supplementary Data 4
Description: List of genes across bins and corresponding Kis values

File Name: Supplementary Data 5
Description: Names of differentially expressed genes related to starvation exit

File Name: Supplementary Data 6
Description: Mass Spectrometry results of Xrn1 WT and mutant interactome

File Name: Supplementary Data 7
Description: Gating strategy of the Flow cytometry experiment
