# Supplementary material for: RNA-controlled nucleocytoplasmic shuttling of mRNA decay factors regulates mRNA synthesis and a novel mRNA decay pathway
Source: Nat Commun. 2022 Nov 23;13:7184. doi: 10.1038/s41467-022-34417-z (PMC9684461; doi:10.1038/s41467-022-34417-z)
Supplement: Supplementary file 10 — Supplementary Data 7 [file 41467_2022_34417_MOESM10_ESM.pdf]

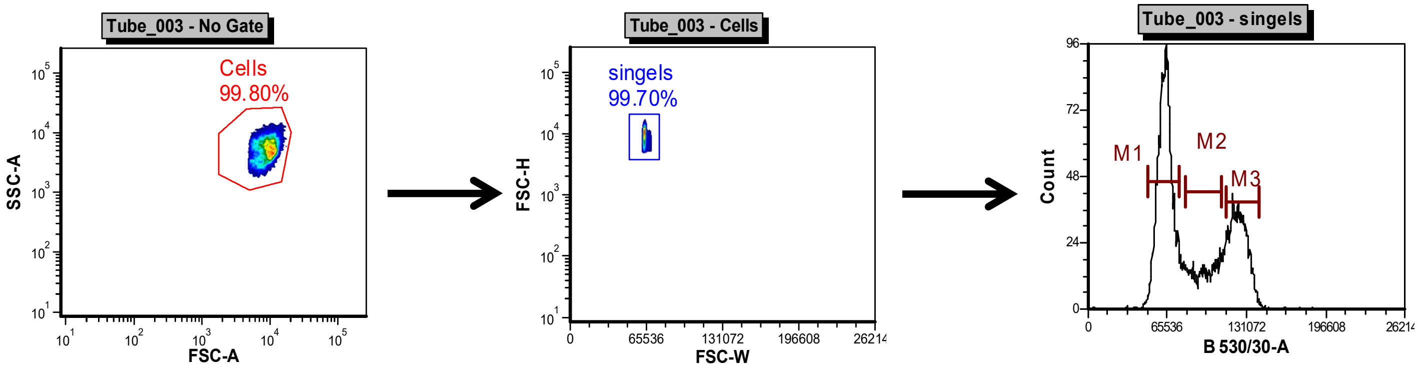

Supplementary Data 7. Gating strategy. Individual budding yeast cells was distinguished from debris and aggregates by examining FSC-A vs SSC-A, followed by FSC-W vs FSC-H was used to exclude doublets
