# Supplementary material for: RNA-controlled nucleocytoplasmic shuttling of mRNA decay factors regulates mRNA synthesis and a novel mRNA decay pathway
Source: Nat Commun. 2022 Nov 23;13:7184. doi: 10.1038/s41467-022-34417-z (PMC9684461; doi:10.1038/s41467-022-34417-z)
Supplement: Supplementary file 11 — Source Data [file 41467_2022_34417_MOESM11_ESM.zip › Source data file/Microscopy.pptx]

## Slide 1
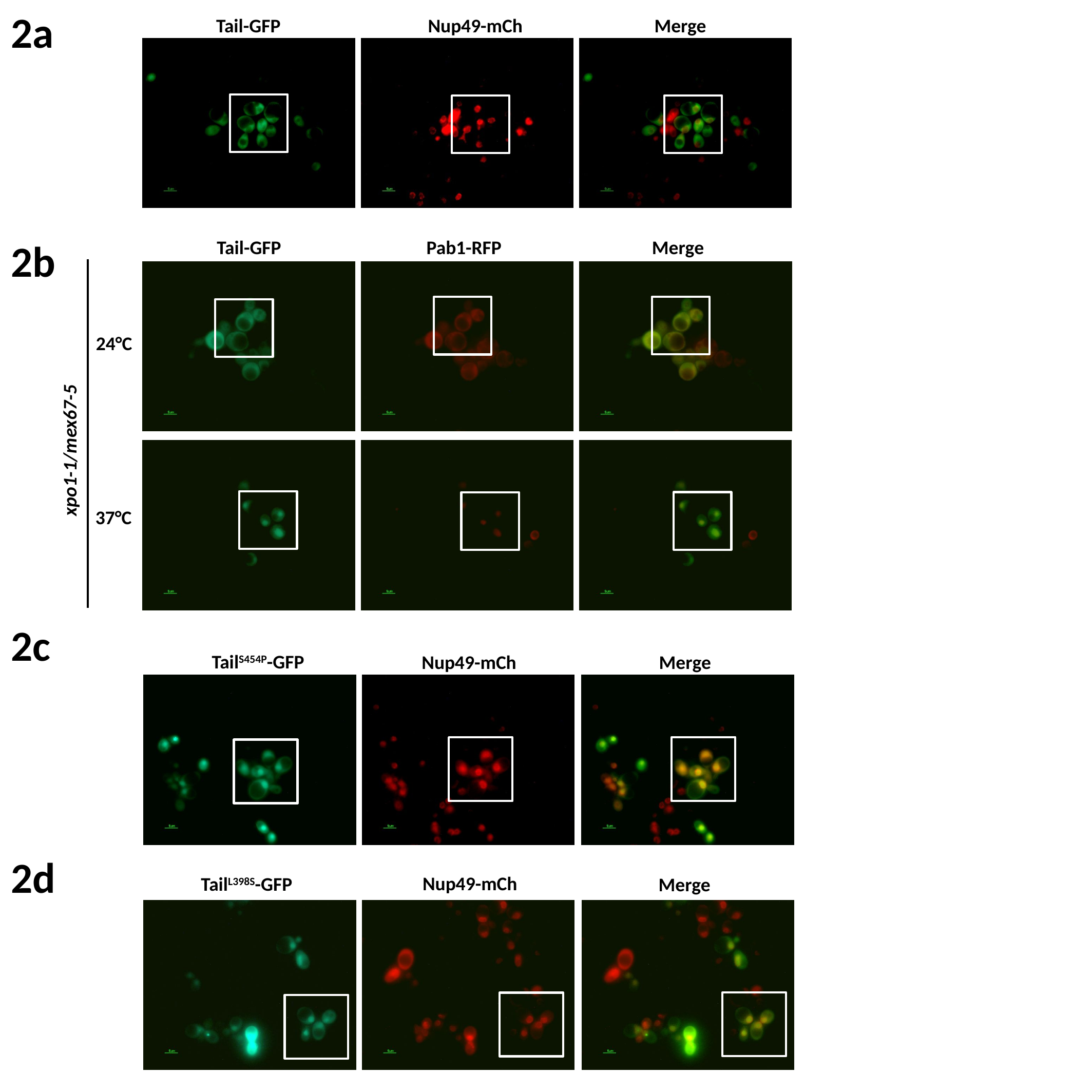

2a
Tail-GFP
Nup49-mCh
Merge
2b
Tail-GFP
Pab1-RFP
Merge
24°C
xpo1-1/mex67-5
37°C
2c
TailS454P-GFP
Merge
Nup49-mCh
2d
Nup49-mCh
TailL398S-GFP
Merge

## Slide 2
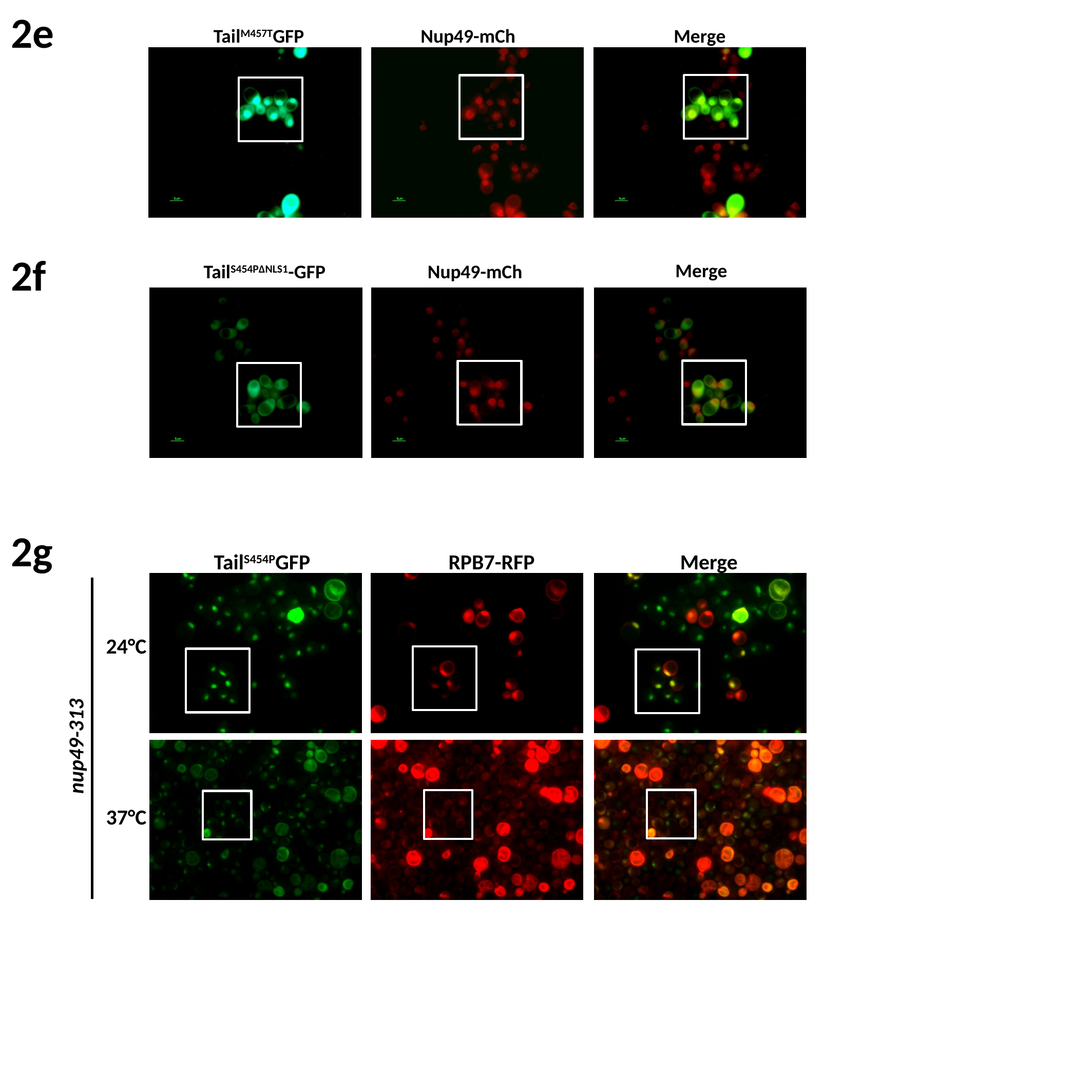

2e
Merge
TailM457TGFP
Nup49-mCh
2f
Merge
TailS454P∆NLS1-GFP
Nup49-mCh
2g
RPB7-RFP
Merge
TailS454PGFP
24°C
nup49-313
37°C

## Slide 3
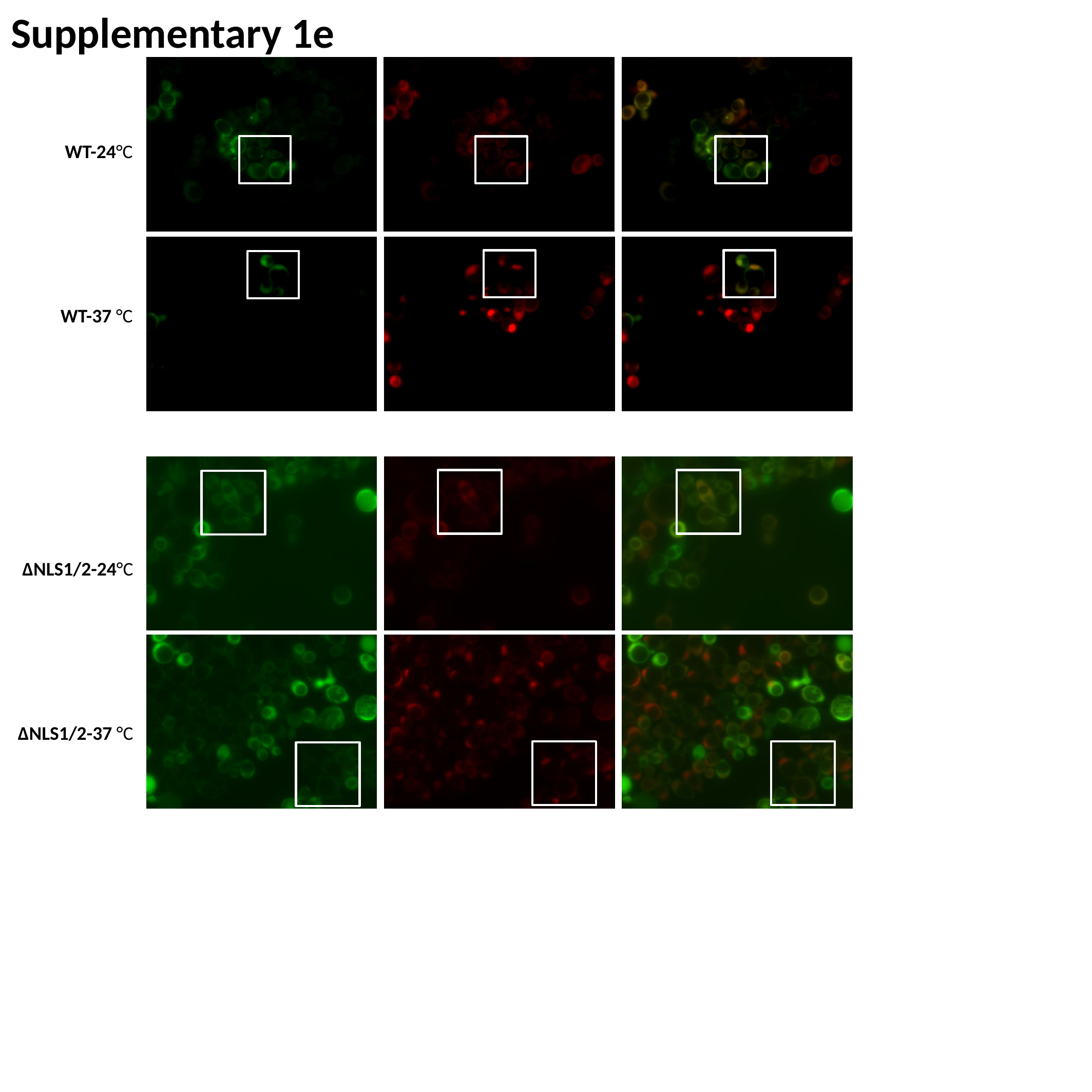

Supplementary 1e
WT-24°C
WT-37 °C
ΔNLS1/2-24°C
ΔNLS1/2-37 °C

## Slide 4
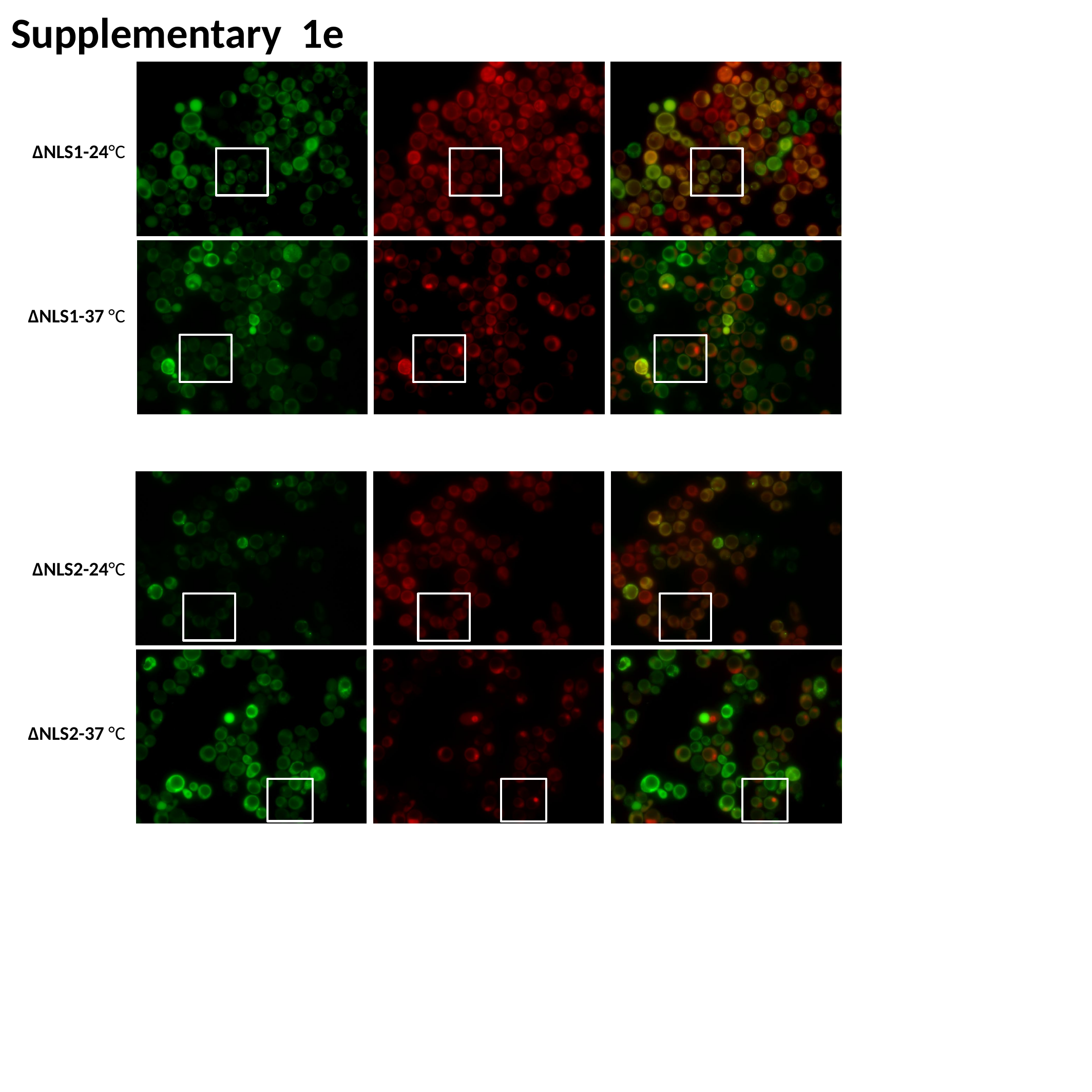

Supplementary 1e
ΔNLS1-24°C
ΔNLS1-37 °C
ΔNLS2-24°C
ΔNLS2-37 °C

## Slide 5
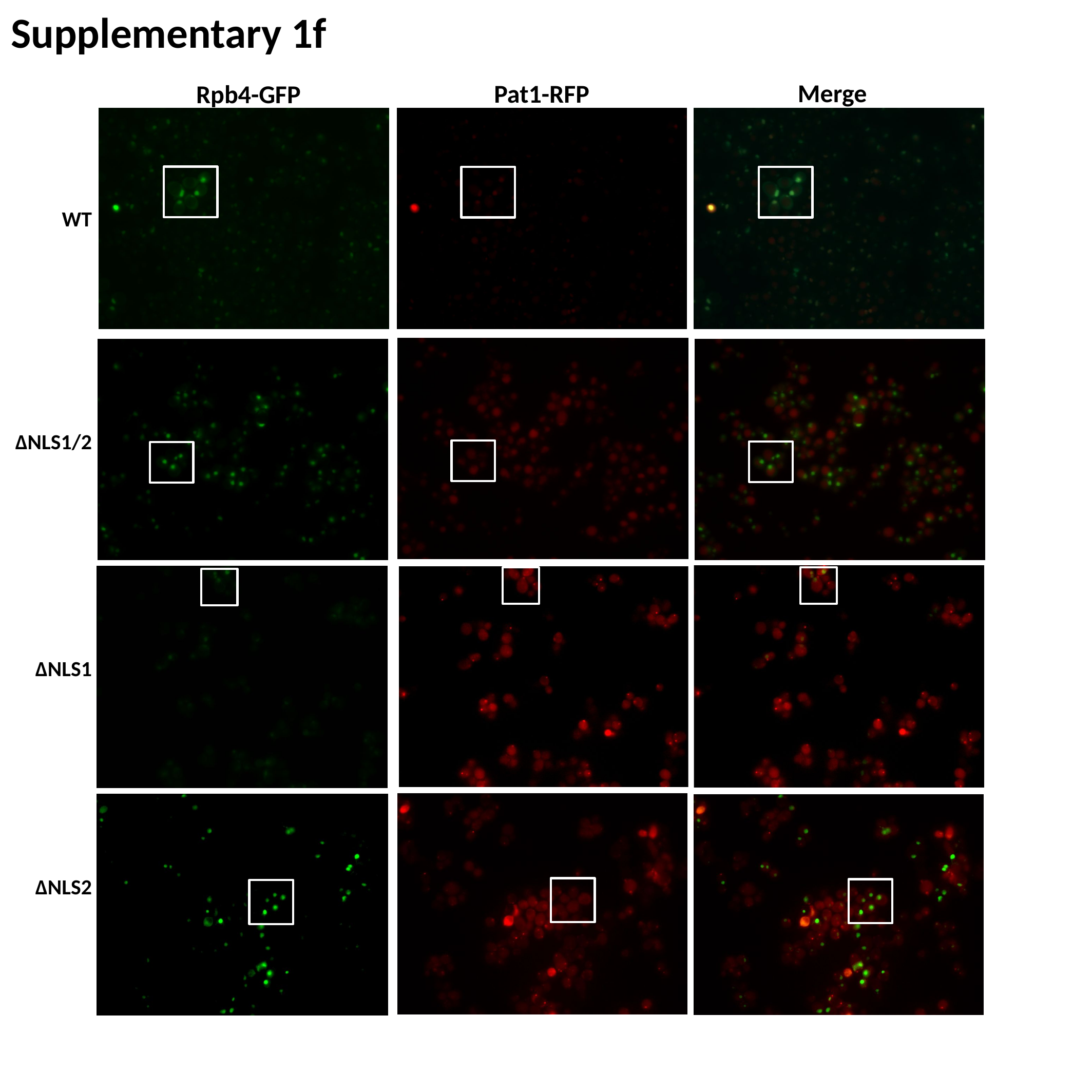

Supplementary 1f
Merge
Pat1-RFP
Rpb4-GFP
WT
∆NLS1/2
∆NLS1
∆NLS2

## Slide 6
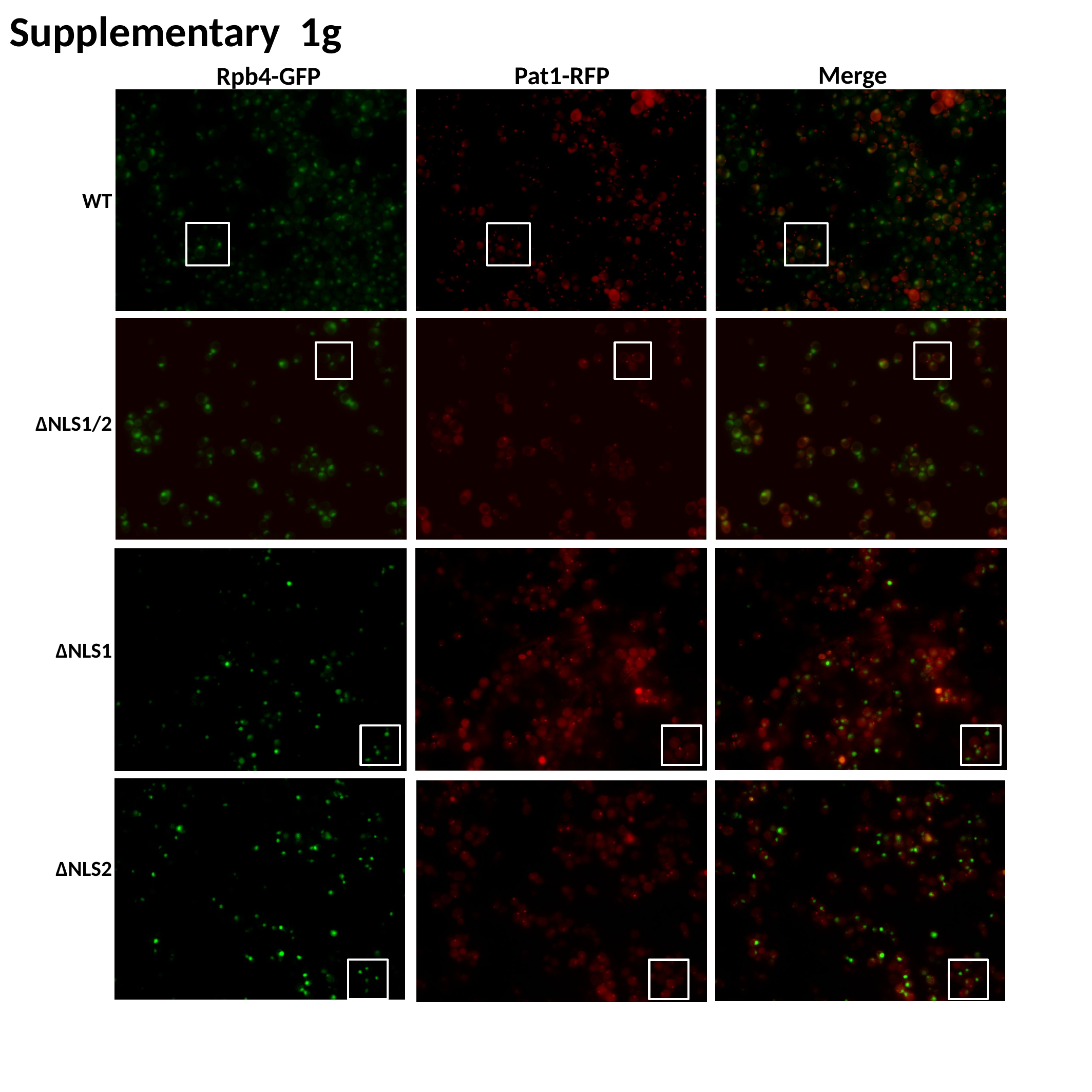

Supplementary 1g
Merge
Pat1-RFP
Rpb4-GFP
WT
∆NLS1/2
∆NLS1
∆NLS2

## Slide 7
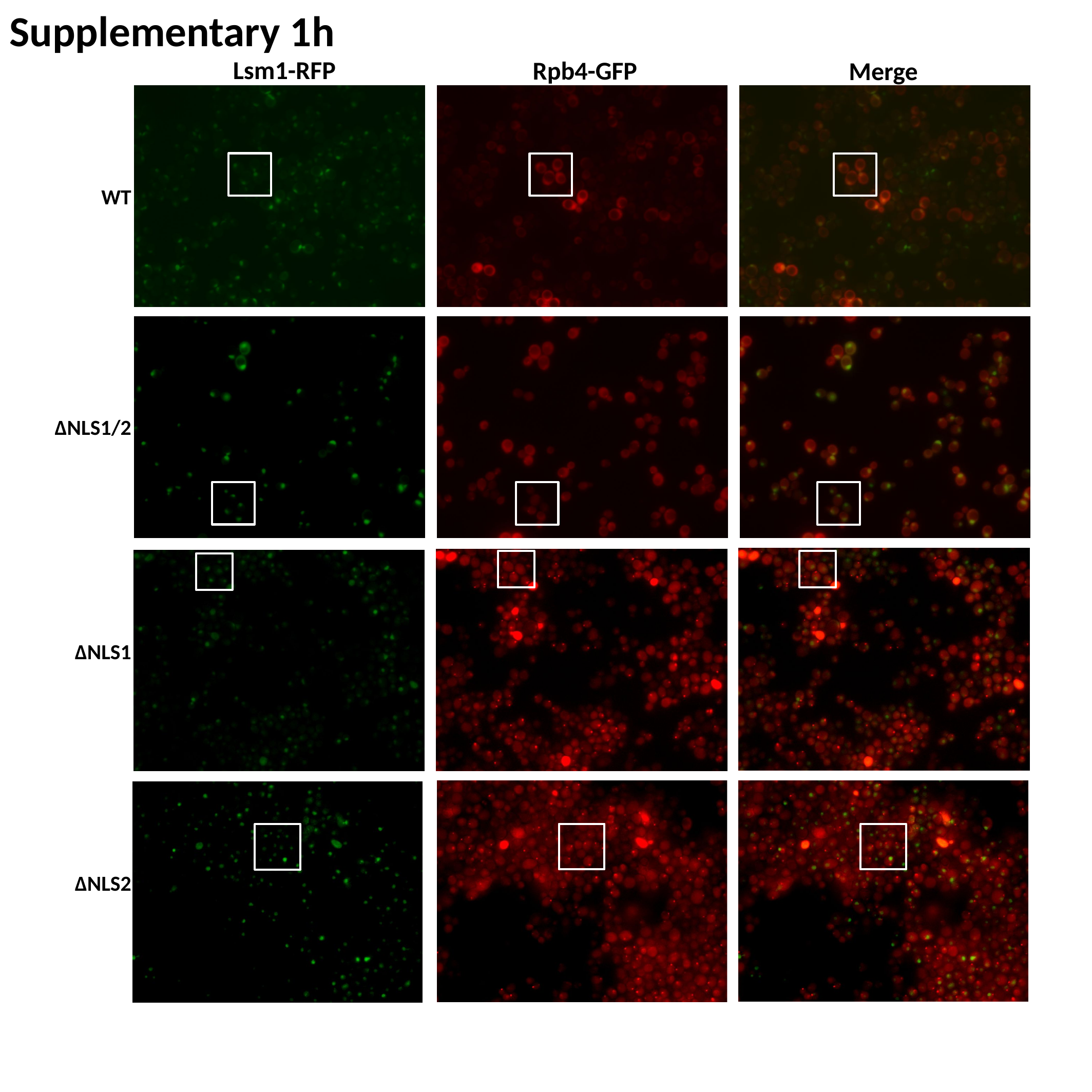

Supplementary 1h
Lsm1-RFP
Rpb4-GFP
Merge
WT
∆NLS1/2
∆NLS1
∆NLS2
