# Supplementary material for: RNA-controlled nucleocytoplasmic shuttling of mRNA decay factors regulates mRNA synthesis and a novel mRNA decay pathway
Source: Nat Commun. 2022 Nov 23;13:7184. doi: 10.1038/s41467-022-34417-z (PMC9684461; doi:10.1038/s41467-022-34417-z)
Supplement: Supplementary file 11 — Source Data [file 41467_2022_34417_MOESM11_ESM.zip › Source data file/Uncropped Raw Blots.pptx]

## Slide 1
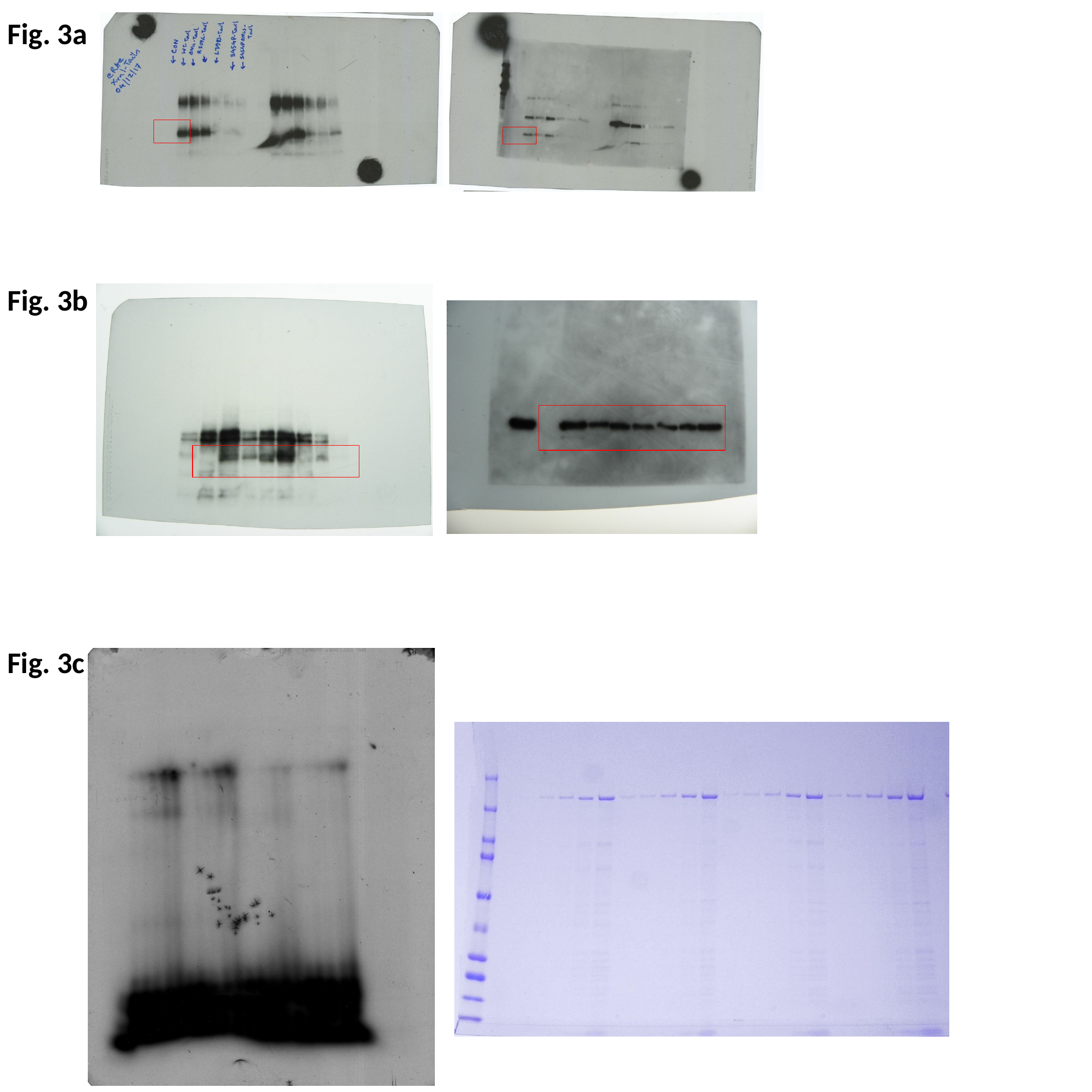

Fig. 3a
Fig. 3b
Fig. 3c

## Slide 2
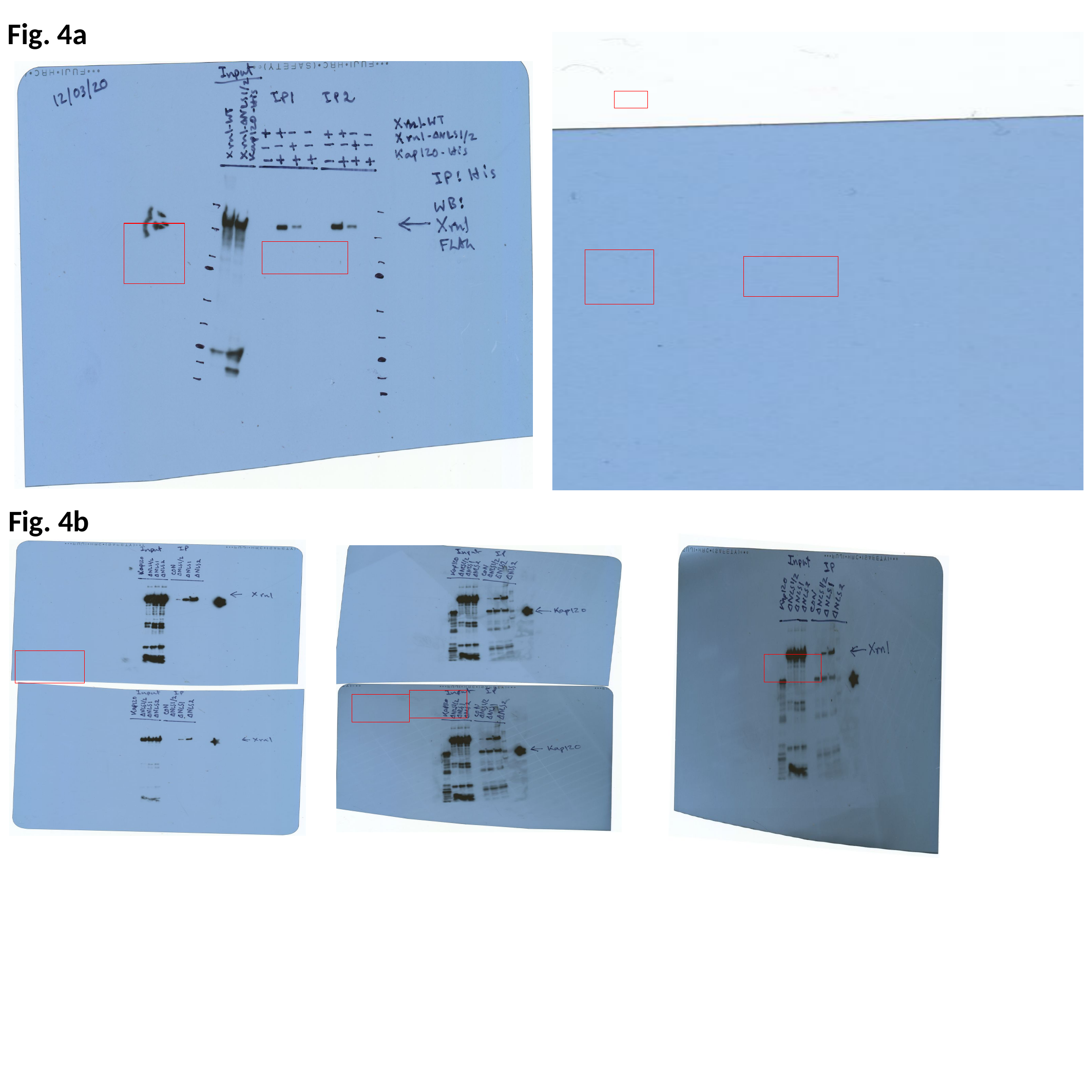

Fig. 4a
Fig. 4b

## Slide 3
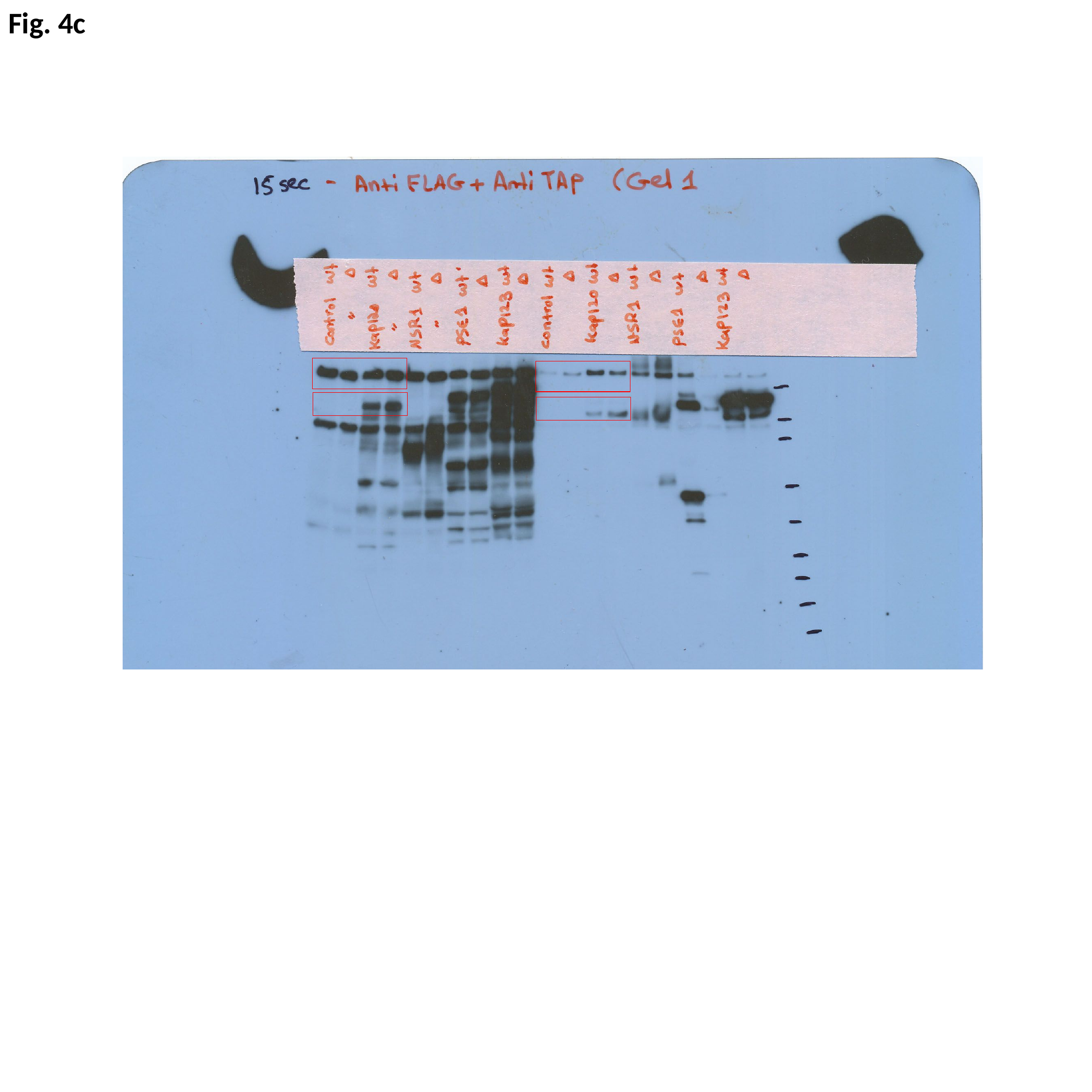

Fig. 4c

## Slide 4
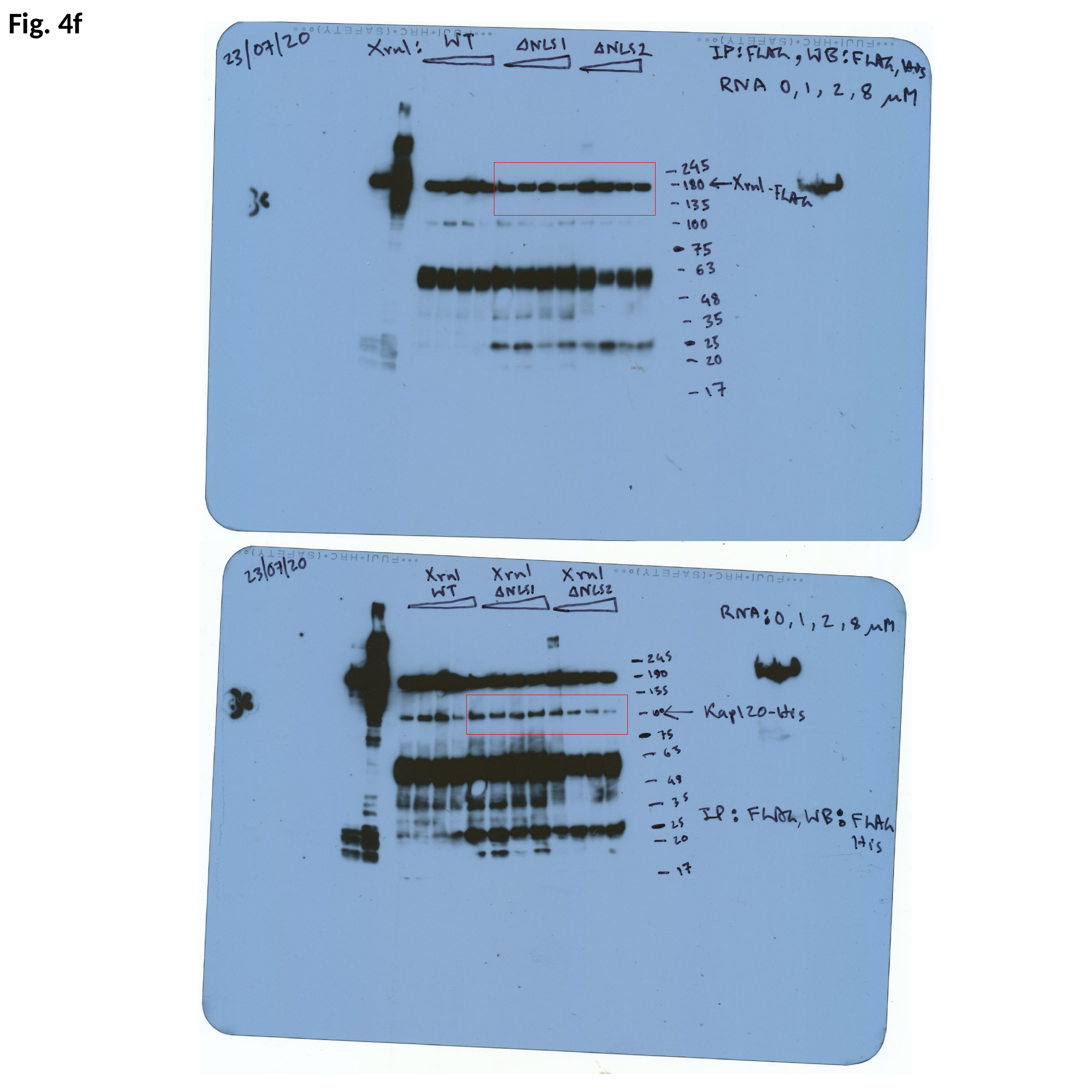

Fig. 4f

## Slide 5
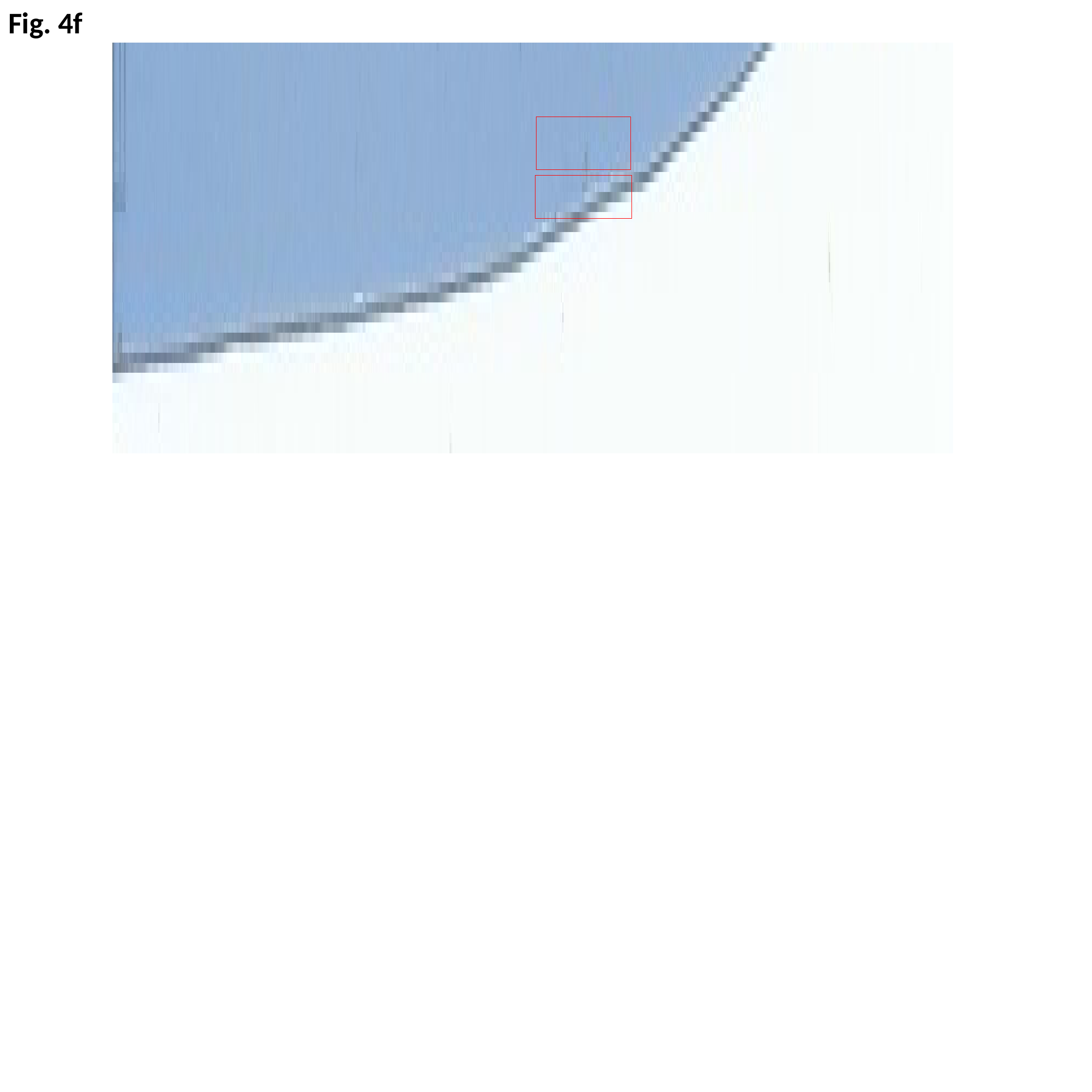

Fig. 4f

## Slide 6
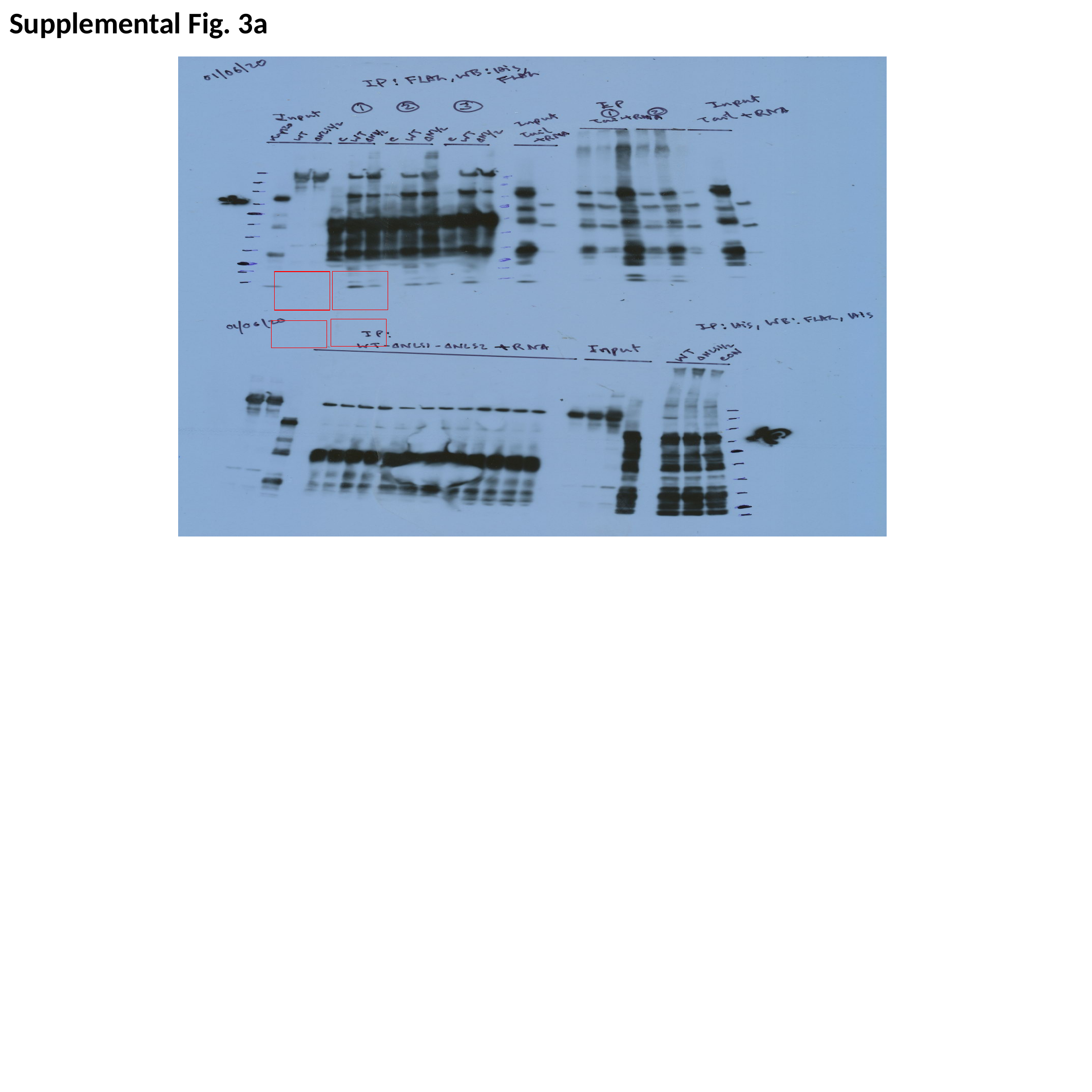

Supplemental Fig. 3a

## Slide 7
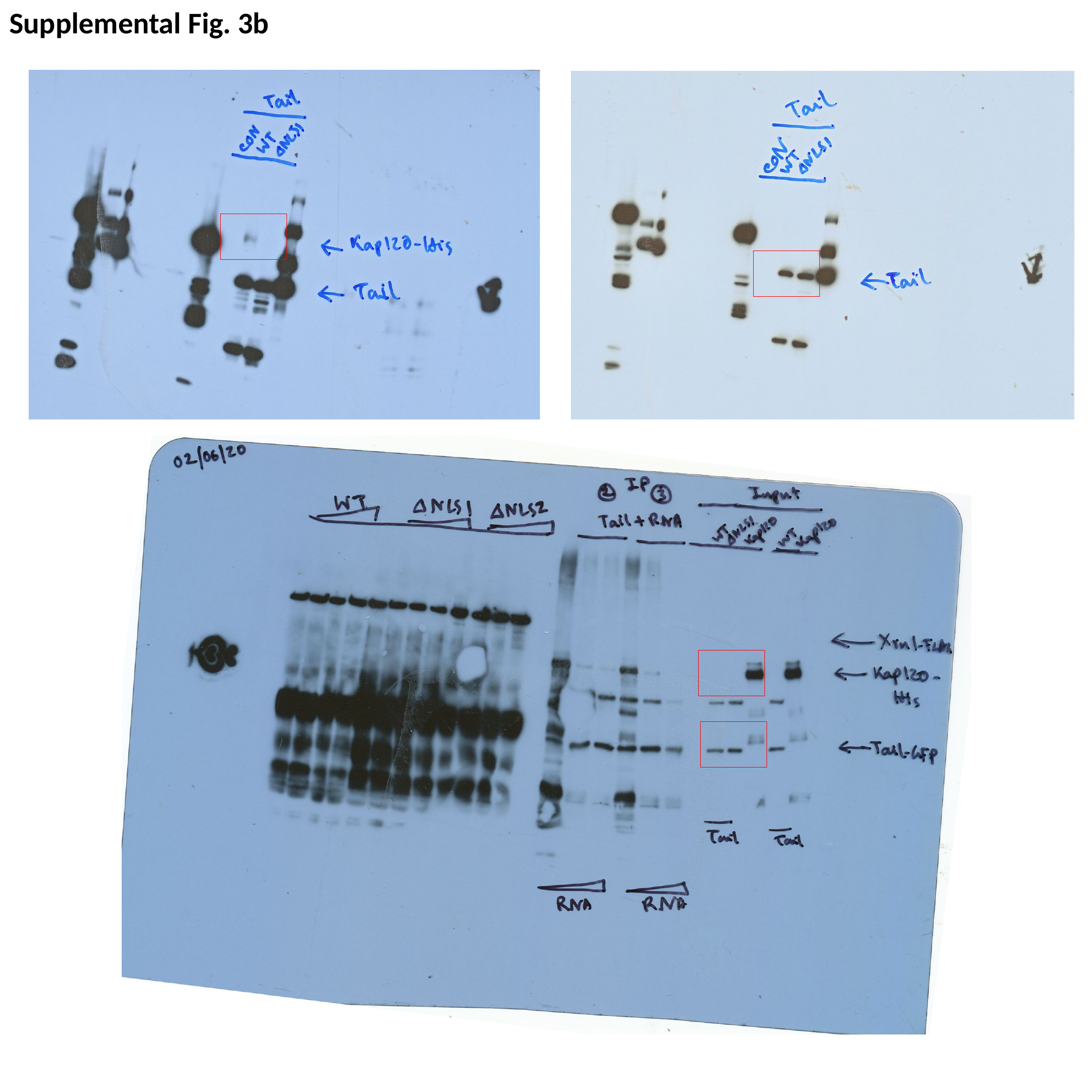

Supplemental Fig. 3b

## Slide 8
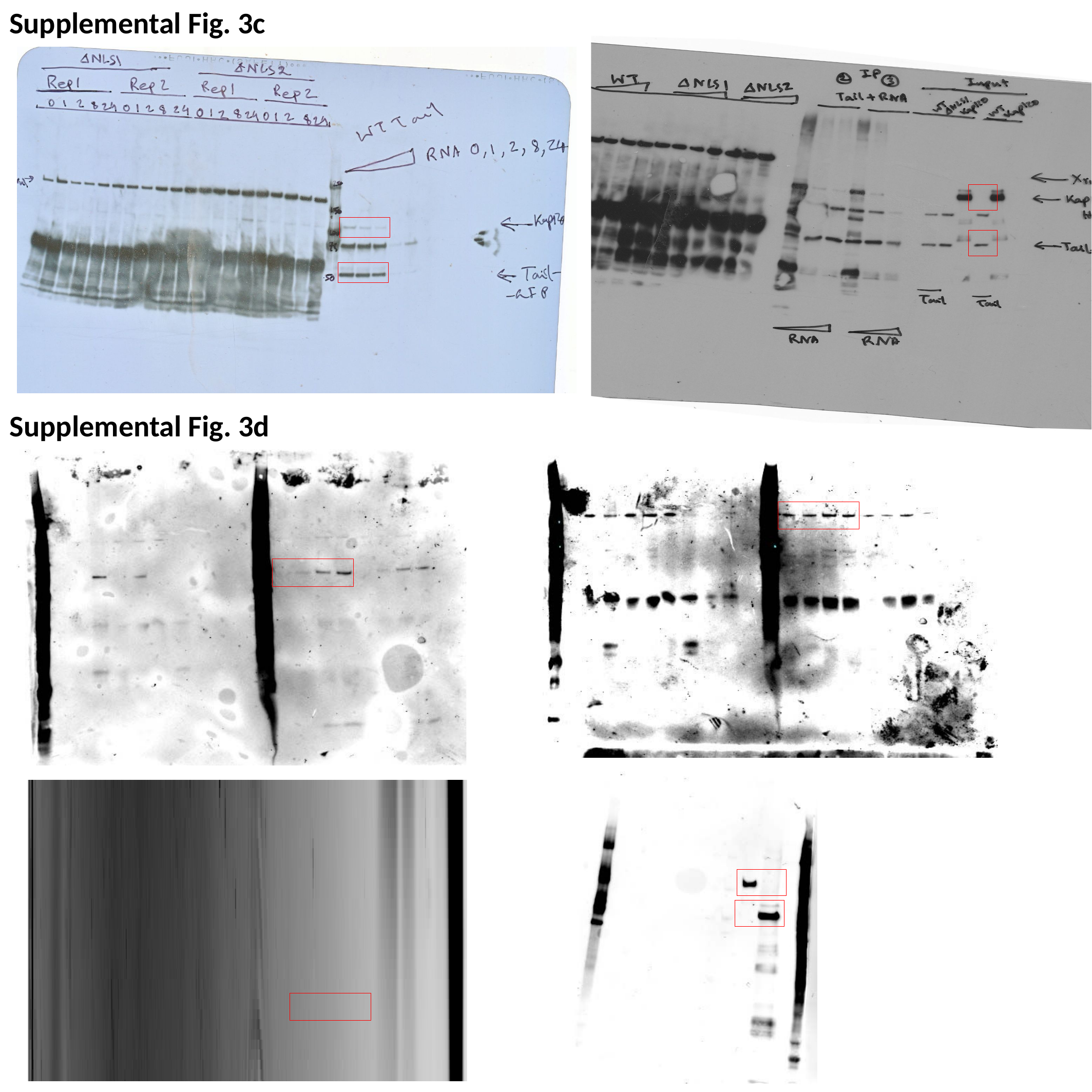

Supplemental Fig. 3c
Supplemental Fig. 3d

## Slide 9
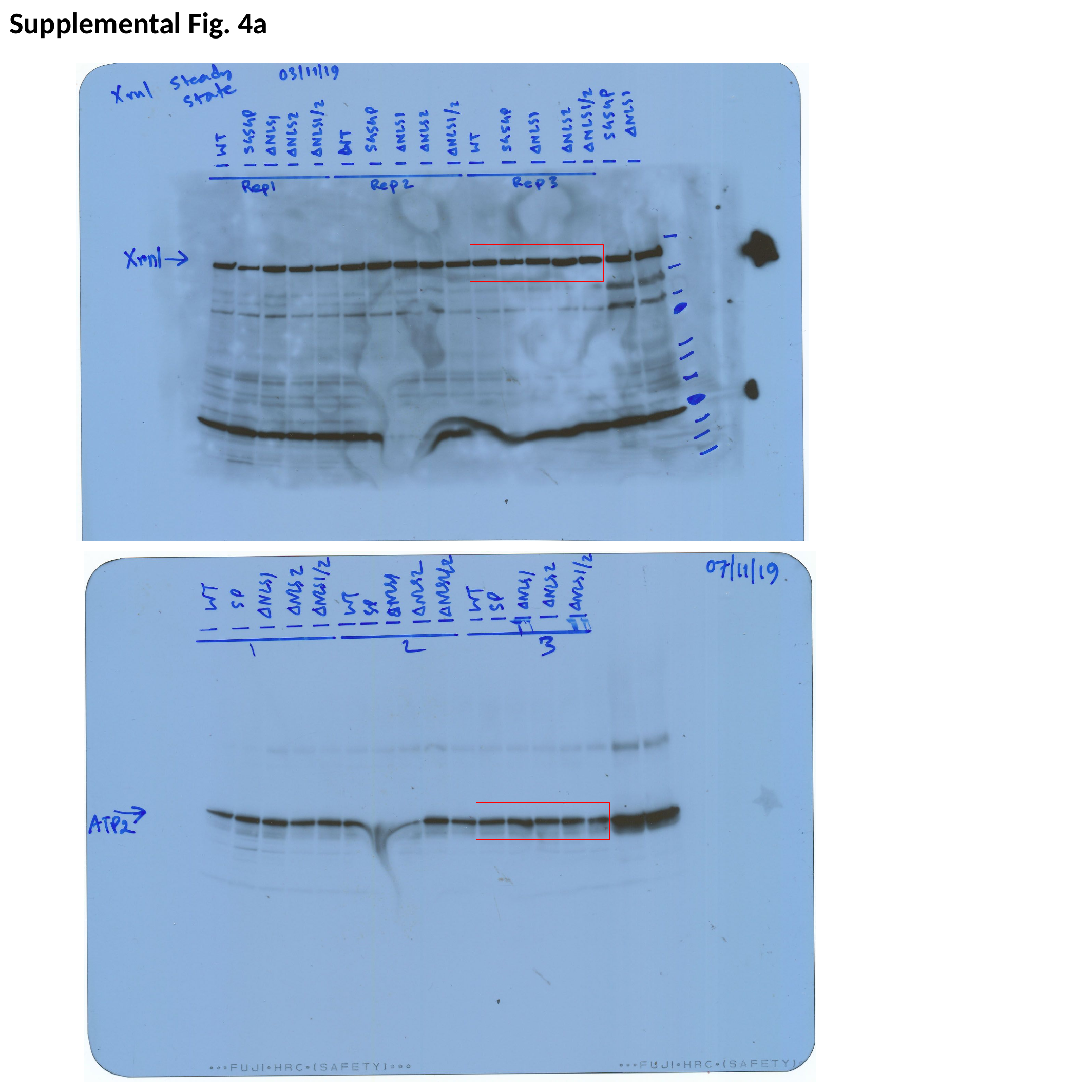

Supplemental Fig. 4a

## Slide 10
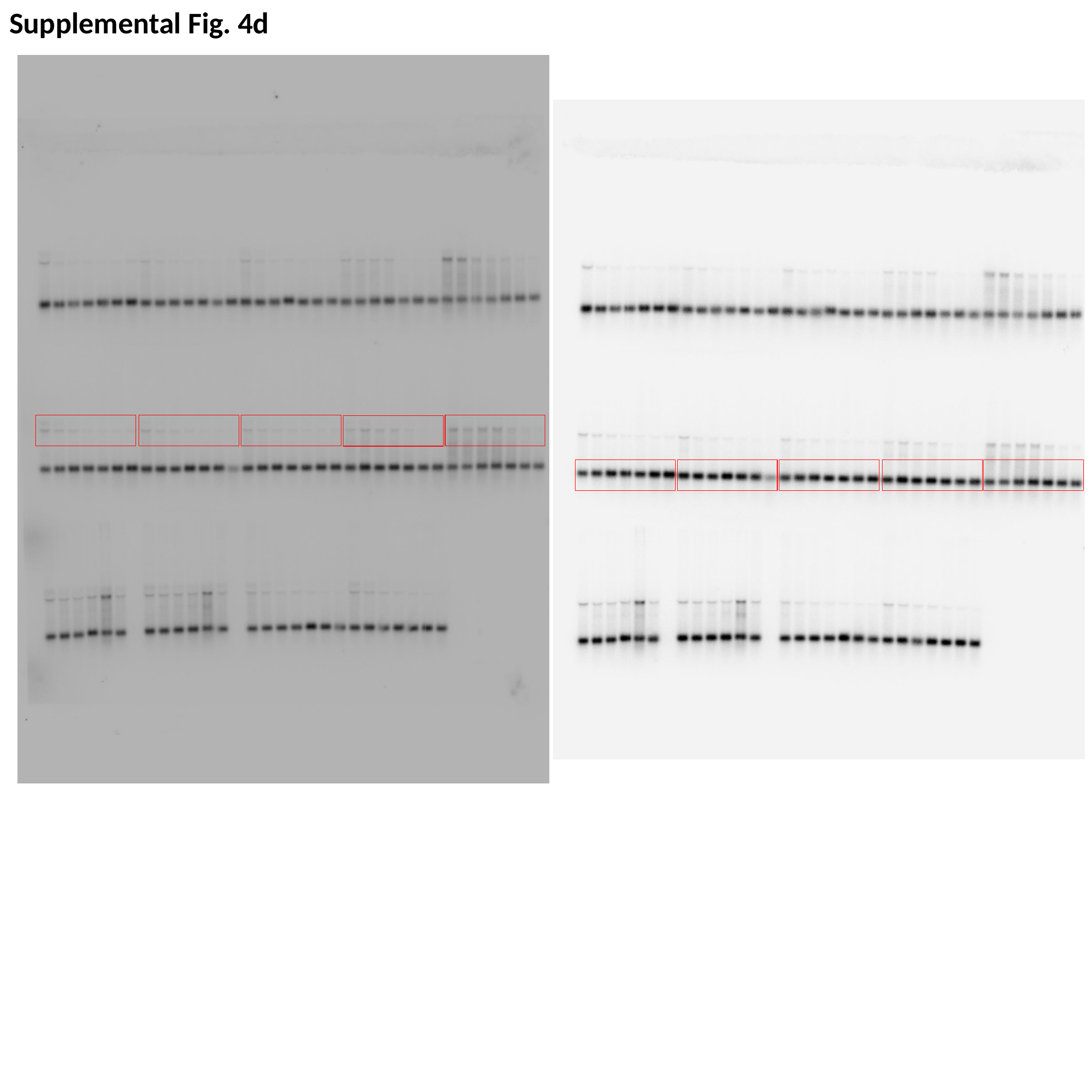

Supplemental Fig. 4d

## Slide 11
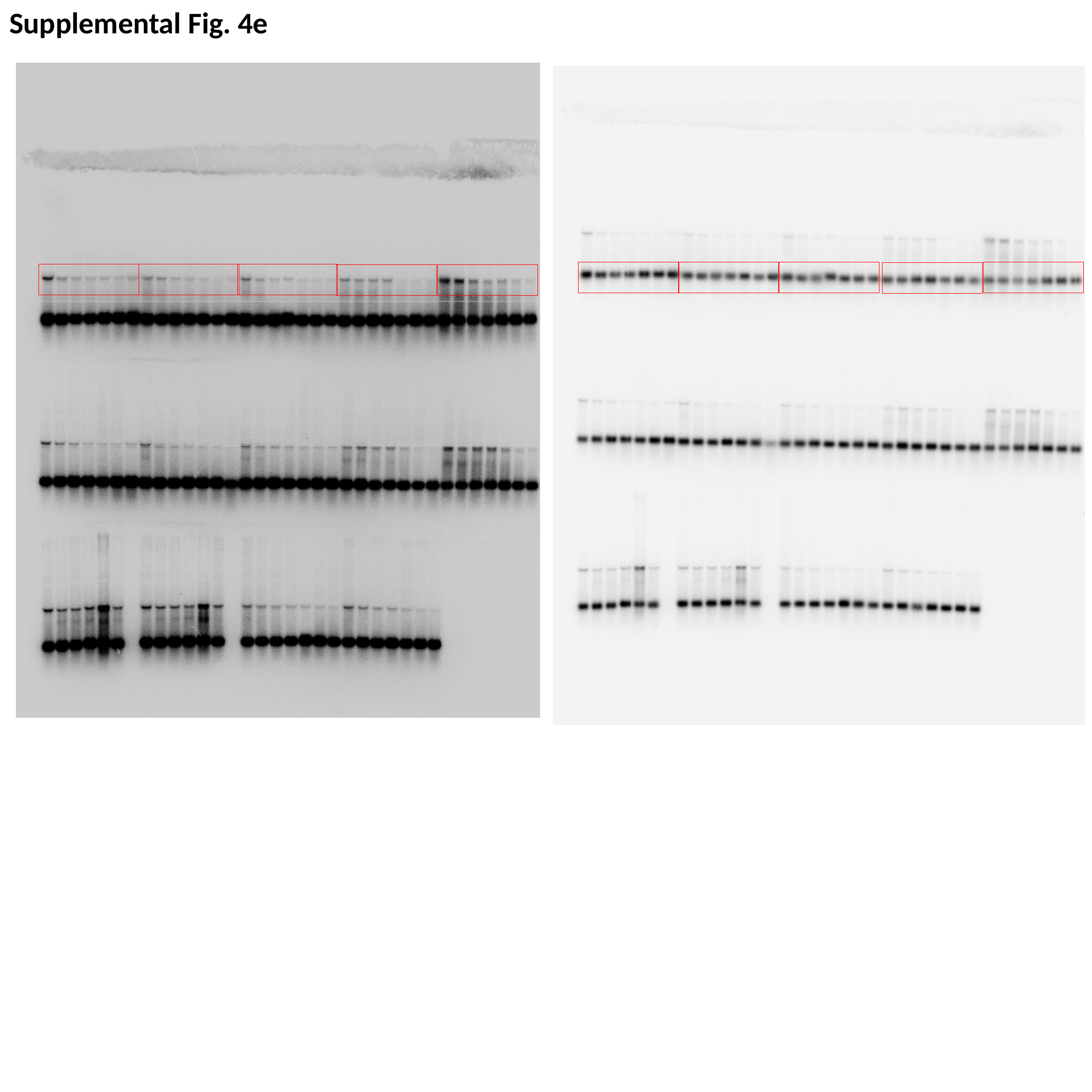

Supplemental Fig. 4e

## Slide 12
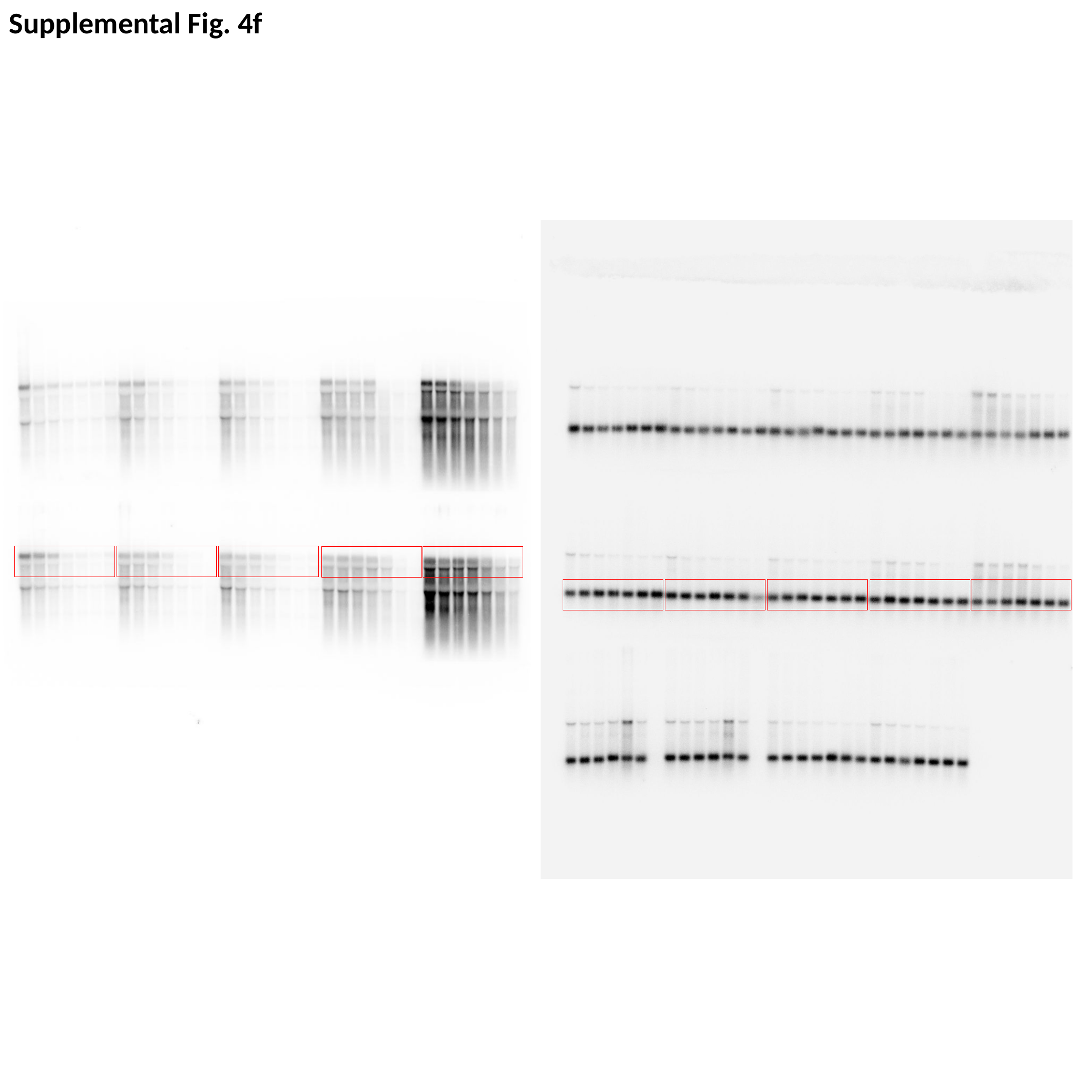

Supplemental Fig. 4f

## Slide 13
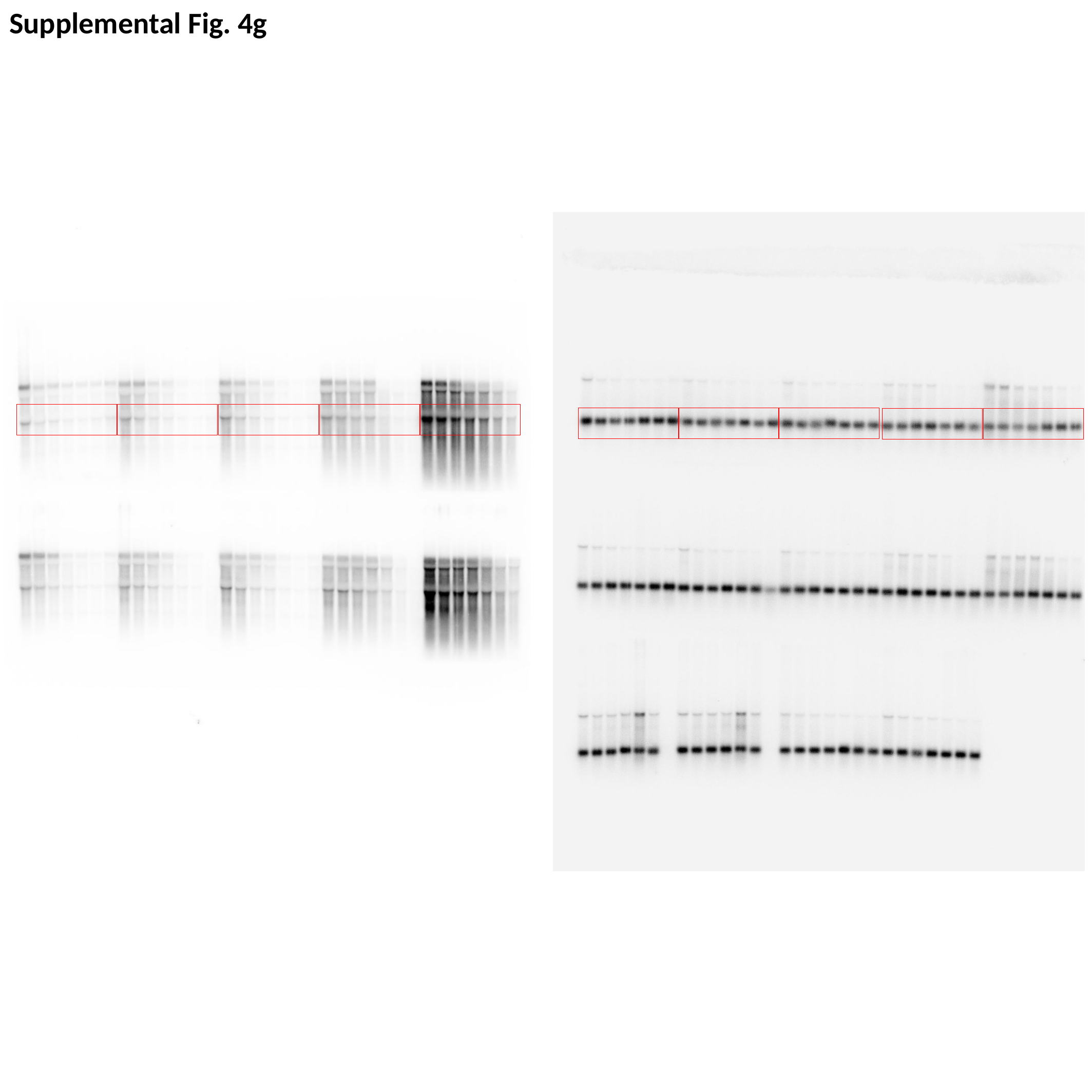

Supplemental Fig. 4g
